# Watershed‐scale effects of tallgrass prairie reconstruction: 30‐Year trends in streamflow, nitrate, and sediment in Walnut Creek, Iowa

**DOI:** 10.1002/jeq2.70174

**Published:** 2026-04-05

**Authors:** Elliot S. Anderson, Keith E. Schilling, Kevin J. Cole, Kenneth M. Wacha

**Affiliations:** ^1^ Iowa Geological Survey University of Iowa Iowa City Iowa USA; ^2^ USDA‐ARS National Laboratory for Agriculture and the Environment Ames Iowa USA

## Abstract

Converting agricultural landscapes to native ecosystems has long been known to improve water quality, especially in US Midwest basins struggling with nutrient and sediment pollution. While the benefits of restoration have been well documented at field scales, few restoration efforts have been substantial enough to be detected at a larger watershed scale. Walnut Creek in central Iowa is a notable exception, undergoing conversion of 45% of cropland in the downstream half of the basin to tallgrass prairie at the Neal Smith National Wildlife Refuge. This study examined the influence of this large‐scale prairie reconstruction on streamflow, nitrate, and sediment in Walnut Creek (1995–2024) using monitoring records upstream and downstream of the restoration. Mean annual streamflow yield and baseflow index were found to be lower in the prairie portion of the basin (264 mm/year and 0.58) than in its unaltered upstream counterpart (292 mm/year and 0.66). Likewise, nitrate and suspended sediment concentration (SSC) levels exhibited greater reductions within the prairie portion of the basin. Nitrate yields and flow‐weighted concentrations decreased at rates of −0.370 kg/ha/year and −0.131 mg/L/year, respectively, while SSC decreases were −48.8 kg/ha/year and −17.0 mg/L/year. These results align with the hypothesized effects of converting cropland to tallgrass prairie and suggest that restoration efforts have had an appreciable impact on Walnut Creek's streamflow and water quality. The study highlights the benefits of long‐term monitoring for quantifying changes in flow and water quality driven by land cover change, as improvements can be difficult to detect due to interannual rainfall variability.

## Core Ideas

- Walnut Creek in central Iowa provides a rare opportunity to assess basin‐scale grassland restoration on water quality.
- Converting cropland to prairie reduced Walnut Creek's streamflow and baseflow index.
- Prairie conversion led to significant reductions in nitrate and sediment in Walnut Creek.
- Long‐term monitoring is needed to account for climactic variations in trend analyses.

## Plain Language Summary

The Neal Smith National Wildlife Refuge (NSNWR) in central Iowa is a rare example of a location where large amounts of cropland have been converted to native prairie. The NSNWR was founded in the early 1990s and provides an opportunity to study the long‐term impacts of land use change on streamflow and water quality. Streamflow, nitrate, and sediment were measured in Walnut Creek both upstream and downstream of the NSNWR over the past 30 years. We estimated yearly streamflow, nitrate, and sediment at these two monitoring sites to explore the impact of the NSNWR. For streamflow, the percentage of water originating from the NSNWR area declined. For nitrate and sediment (which are two common pollutants), the percentage coming from the NSNWR area also declined, though this decline was more significant for nitrate than for sediment. These results show that the benefits of large prairie restorations can be detected at the watershed scale.

**Abbreviations**
ARS: Agricultural Research Service
BFI: baseflow index
FW conc: flow‐weighted concentrations
NSNWR: Neal Smith National Wildlife Refuge
SSC: suspended sediment concentration
USGS: United States Geological Survey

## 1 INTRODUCTION

Land use change strongly influences watershed hydrology (Chawla & Mujumdar, 2015) and surface water quality (Tong & Chen, 2002). In the US Midwest, conversion of native prairies, savannas, and forests to row crop agriculture has increased runoff, altered groundwater recharge, and enhanced nutrient and sediment delivery to waterbodies (Brown et al., 2000; McCorvie & Lant, 1993; Rhemtulla et al., 2007). While this transformation has greatly boosted agricultural outputs, it has also degraded aquatic habitat and impaired downstream waterbodies (Jaynes et al., 2010).

In response to these environmental concerns, efforts have been made to remediate waterborne pollutants through conservation (Prokopy et al., 2019). One established strategy involves taking agricultural land out of production and returning it to a more native ecosystem (Camill et al., 2004). Many benefits of native restorations are well‐substantiated within environmental theory and have been demonstrated in several studies focused on land parcels or small subcatchments (Helmers et al., 2012; Larson et al., 2013). For example, prairie restorations are known to reduce nutrient pollution through their removal of agricultural sources (Christianson et al., 2018), immobilization of waterborne nutrients (Kemp & Dodds, 2002), and conversion of bioavailable nutrients to inert chemical forms (Skinner, 2022). Many native landscapes offer the additional benefit of lessening flood risk (Amado et al., 2018).

However, due to economic and political incentives, large‐scale conversion of agricultural land to native ecosystems remains rare (T. A. Jones, 2018), with most efforts encompassing <100 ha (Kimball et al., 2015). Consequently, detecting the impacts of conversion efforts at large watershed scales has remained elusive (Cheng et al., 2022; Giri & Qiu, 2016). Opportunities to perform such studies are scarce, not only because they require identifying a watershed where a nontrivial amount of its landscape has been restored, but also because the waterway must be sufficiently monitored. Therefore, tangible reports of water quality improvements attributable to land use change are uncommon (Wijesiri et al., 2018), and many evaluations have proven inconclusive due to the limited spatial extent of upstream landscape conversions or limited temporal extent of the monitoring (Johnes & Heathwaite, 1997).

In this study, we investigated temporal streamflow and water quality trends in an Iowa watershed that may provide a “best‐case” scenario for such an analysis. Since the mid‐1990s, approximately 30% of Walnut Creek's area has been converted from cropland into native prairie, and water quality monitoring recently concluded its 30th year. The rare, long‐term monitoring effort provides a timescale sufficient for characterizing water quality (Hirsch et al., 2015) and hydrologic trends (Arguez & Vose, 2011). While previous studies have evaluated nutrient and sediment trends within Walnut Creek and suggested positive improvements (Schilling & Spooner, 2006; Schilling et al., 2011; Tomer et al., 2019), they all recommended future monitoring and analyses to more thoroughly document the downstream impact of its land use conversion.

## 2 MATERIALS AND METHODS

### 2.1 Site description and history

**FIGURE 1:**
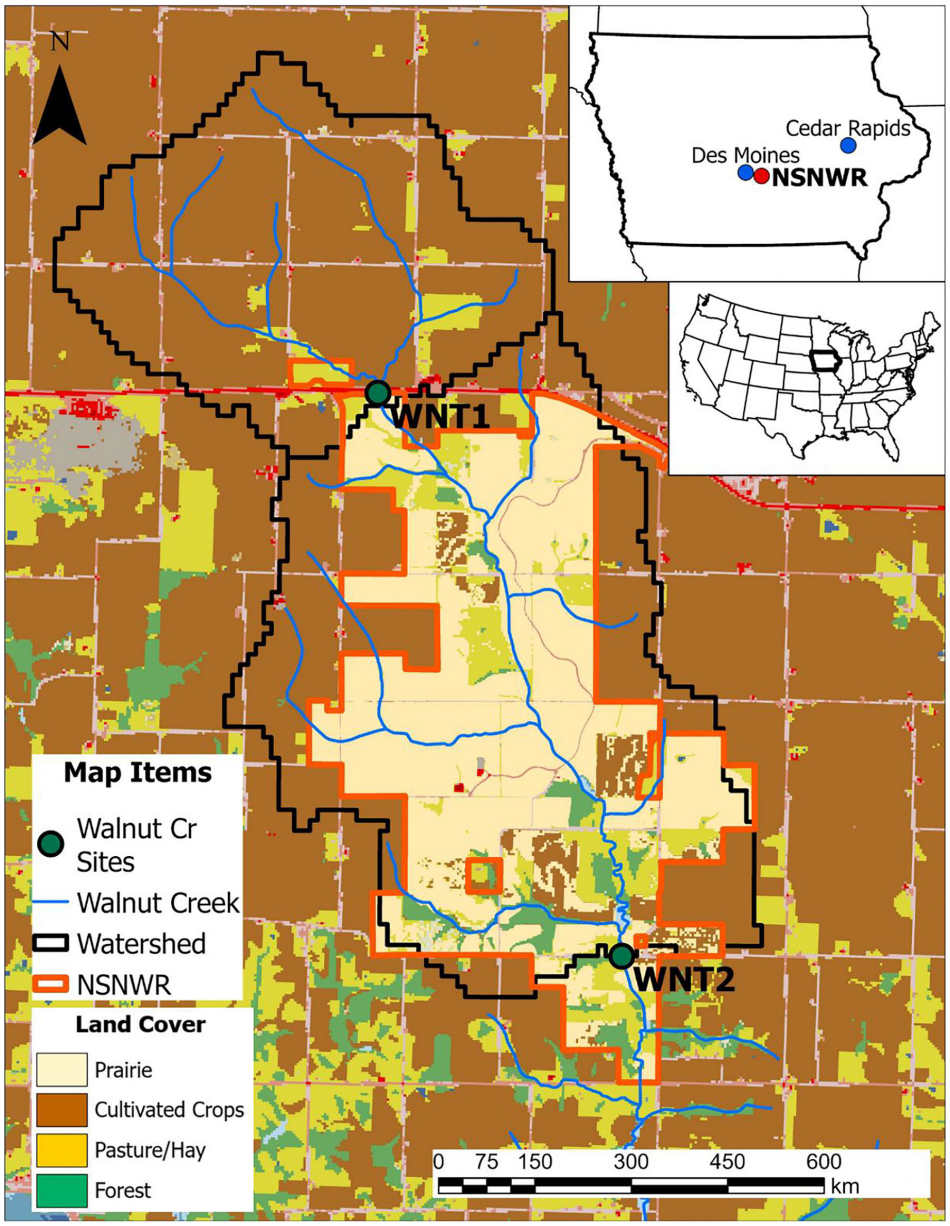
Location map highlighting the Neal Smith Wildlife Refuge (NSNWR) area, including its watershed, monitoring sites, and land cover (circa 2024). WNT1, upstream monitoring site along Walnut Creek; WNT2, downstream monitoring site along Walnut Creek.

This study focused on the Walnut Creek watershed (HUC12 071000081505), a 7900‐ha basin in central Iowa (Figure 1). Walnut Creek is a perennial stream that flows from north to south before discharging into the much larger Des Moines River. For much of its history, this watershed was unremarkable, with land use, climate, and geologic features very similar to those of most agricultural catchments in Iowa (Schilling & Drobney, 2014). However, this changed in 1990 with the founding of the Neal Smith National Wildlife Refuge (NSNWR). The NSNWR is a federal wildlife refuge located in the southern portion of the Walnut Creek basin, whose primary goal is restoring the native prairie and savanna ecosystems that once characterized much of Iowa.

While small portions of oak savanna (<200 ha) have been maintained by rehabilitating Walnut Creek's riparian corridor, the vast majority of the NSNWR's work has centered on converting conventional cropland to native tallgrass prairie. Prairie restoration began in 1995, and >70% of the refuge's land was converted by the mid‐2000s. Additional minor restorations have continued over the past two decades, and today, nearly 1700 ha of cropland has been converted to tallgrass prairie (Figure 1). These efforts have resulted in Walnut Creek being a rare example of a basin with a significant amount of its tributary area restored to its native condition (Schilling & Drobney, 2014).

Because of its unique land use history, Walnut Creek has served as a valuable testbed for numerous studies examining the impacts of native landscape restoration on the environment—especially water quality (Schilling & Spooner, 2006; Schilling & Thompson, 2000; Tomer et al., 2019). In 1995, the Iowa Department of Natural Resources (IDNR) established two monitoring stations along Walnut Creek's main stem: (1) WNT1 (upstream monitoring site along Walnut Creek) and (2) WNT2 (downstream monitoring site along Walnut Creek). These two sites are located just upstream (WNT1) and downstream (WNT2) of the refuge (Figure 1). The IDNR continued monitoring until 2005, when efforts were paused and then reestablished by the Agricultural Research Service (ARS) in 2007. The ARS has continued monitoring to present day, resulting in a robust record of streamflow and water quality measurements spanning 30 years (1995–2024).

**FIGURE 2:**
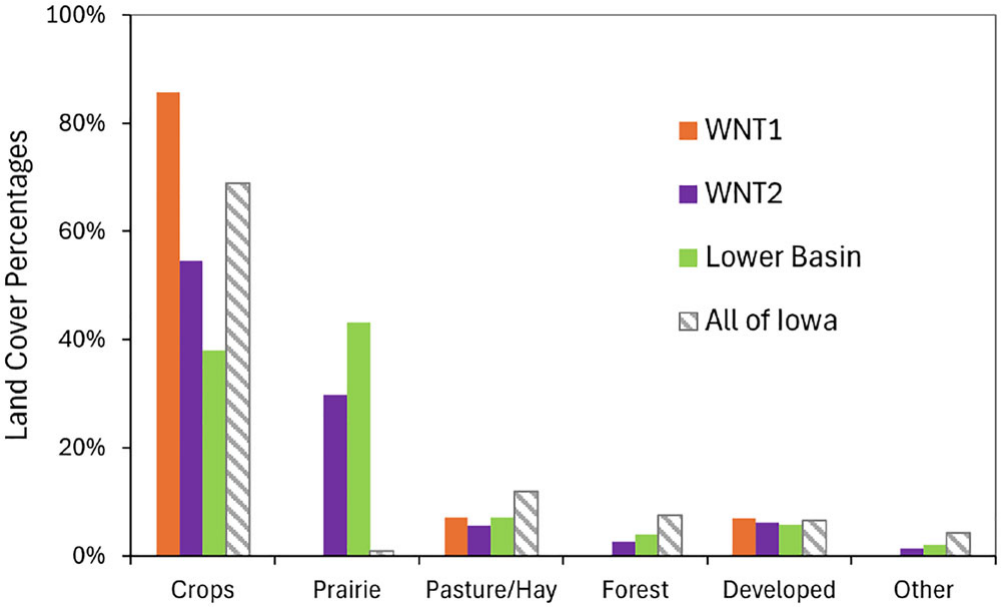
Summary of current land cover within the Walnut Creek watershed, organized by monitoring site. Land coverages for the entire state of Iowa are provided as a reference. WNT1, upstream monitoring site along Walnut Creek; WNT2, downstream monitoring site along Walnut Creek.

The landscape upstream of WNT1 remains largely agricultural and stands in stark contrast to that of the NSNWR. Because the WNT2 monitoring site contains drainage area from both the NSNWR and the agricultural area above it (which is captured by WNT1), it is often necessary to subtract riverine volumes and loads at WNT1 from those of WNT2 to isolate the portion of the watershed where the land use change occurred. We henceforth refer to the values obtained via this calculation using the label Lower Basin, which is formally defined as follows:

LowerBasin=WNT2−WNT1,

where WNT1 and WNT2 represent water volumes or loads at each monitoring site. The Lower Basin thus refers to the part of the watershed containing the NSNWR. Figure 2 shows the modern (circa 2024) land cover throughout the Walnut Creek watershed. The area upstream WNT1 contains no prairie and ∼85% cropland, while the Lower Basin is comprised of ∼45% prairie and ∼35% cropland.

**Core Ideas**

- Walnut Creek in central Iowa provides a rare opportunity to assess basin‐scale grassland restoration on water quality.
- Converting cropland to prairie reduced Walnut Creek's streamflow and baseflow index.
- Prairie conversion led to significant reductions in nitrate and sediment in Walnut Creek.
- Long‐term monitoring is needed to account for climactic variations in trend analyses.

### 2.2 Hydrology analysis

Streamflow has been consistently monitored at WNT1 and WNT2 using site‐specific rating curves that convert stage measurements to corresponding discharge values (Brakensiek, 1979). Ten‐minute discharge measurements were averaged to daily values by taking the arithmetic mean of sub‐daily observations. A gap in the record occurred in 2006 during the monitoring transition. Daily discharge for 2006 was estimated via linear regression against the nearby United States Geological Survey (USGS) gauge 05485640 following *StreamStats* protocols (Ries et al., 2017). Baseflow separations were performed using the one‐parameter digital filter method (Eckhardt, 2005) to approximate the baseflow index (BFI) at each site, and stream flashiness was calculated using the Richards–Baker flashiness index (Baker et al., 2004). Additionally, streamflow measurements were converted to water yields (mm/day) by dividing each site's daily flow values by its tributary area, and these water yields were summed to generate annual values (mm/year).

**FIGURE 3:**
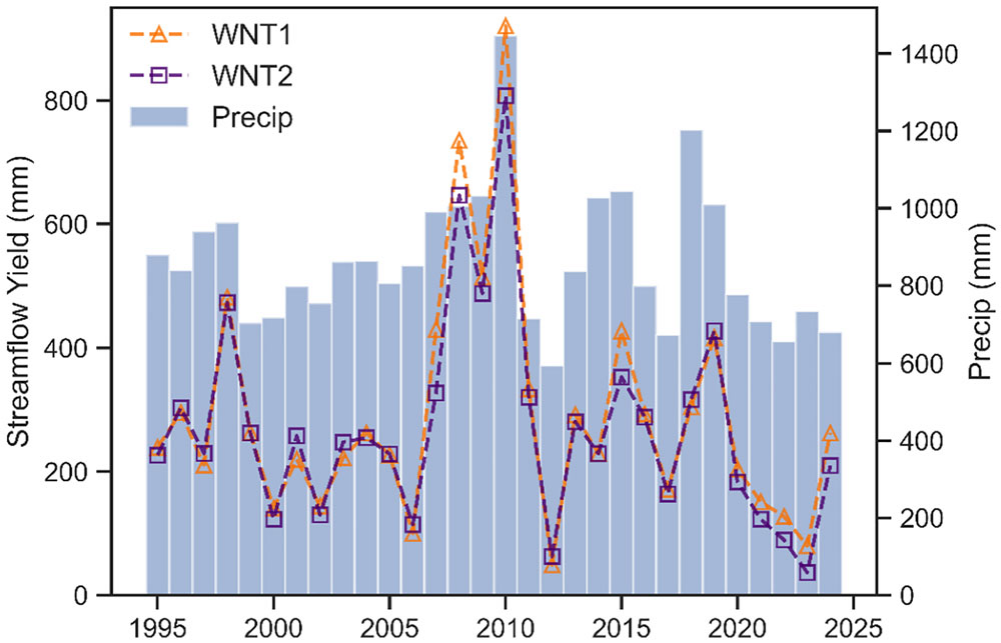
Annual streamflow yield and precipitation at Walnut Creek from 1995 to 2024. Precip, precipitation; WNT1, upstream monitoring site along Walnut Creek; WNT2, downstream monitoring site along Walnut Creek.

**FIGURE 4:**
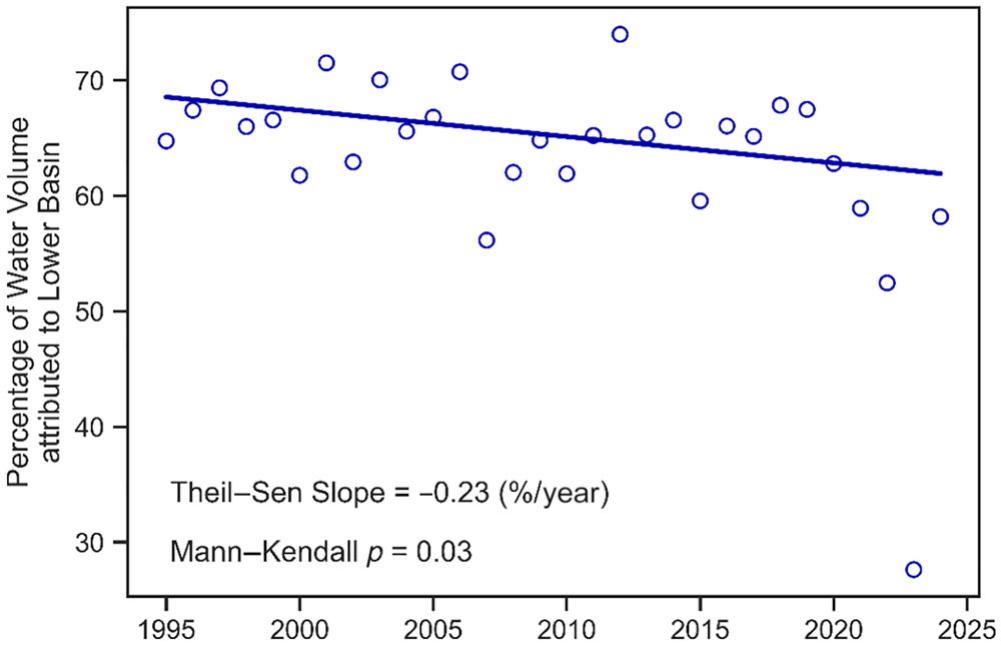
Percentage of annual water volume attributed to the Lower Basin of Walnut Creek from 1995 to 2024. The solid line represents a monotonic trend, with test results provided in the figure text.

Table 1 summarizes several hydrologic statistics for WNT1, WNT2, and the Lower Basin over the entirety of the analysis period, and Figure 3 displays each site's annual water yield and precipitation. Precipitation was obtained from a nearby rain gauge (USC00135992) in Newton, IA. To explore the impact of the NSNWR on water yields over time, we calculated the percentage of the water volume at WNT2 attributed to the Lower Basin (Figure 4). These percentages were found by dividing the water volume from the Lower Basin by WNT2's water volume for each year, thus determining how much of the flow observed at WNT2 originated from the portion of the watershed containing the NSNWR. We explored trends using the Mann–Kendall monotonic trend test that accounts for positive autocorrelation often found within hydrologic datasets (Hamed & Ramachandra Rao, 1998). We estimated the slope of this trend using the Theil–Sen method, which has proven effective at quantifying temporal hydrologic changes (J. Zhou et al., 2023).

**TABLE 1:** Monitoring site information for Walnut Creek and corresponding hydrologic metrics.

Site label	Site full name	Area (ha)	Latitude	Longitude	Annual streamflow yield (mm/year)	Annual baseflow yield (mm/year)	BFI	Flashiness
WNT1	Walnut Creek near Prairie City	1760	41.60085	−93.29599	292	191	0.66	0.40
WNT2	Walnut Creek near Vandalia	5260	41.53703	−93.25897	273	166	0.61	0.48
Lower Basin	Portion of Basin containing NSNWR	3500			264	154	0.58	

*Note*: The streamflow, baseflow, BFI, and flashiness values span the entire study duration (1995–2024).

Abbreviations: BFI, baseflow index; NSNWR, Neal Smith Wildlife Refuge; WNT1, upstream monitoring site along Walnut Creek; WNT2, downstream monitoring site along Walnut Creek.

**FIGURE 5:**
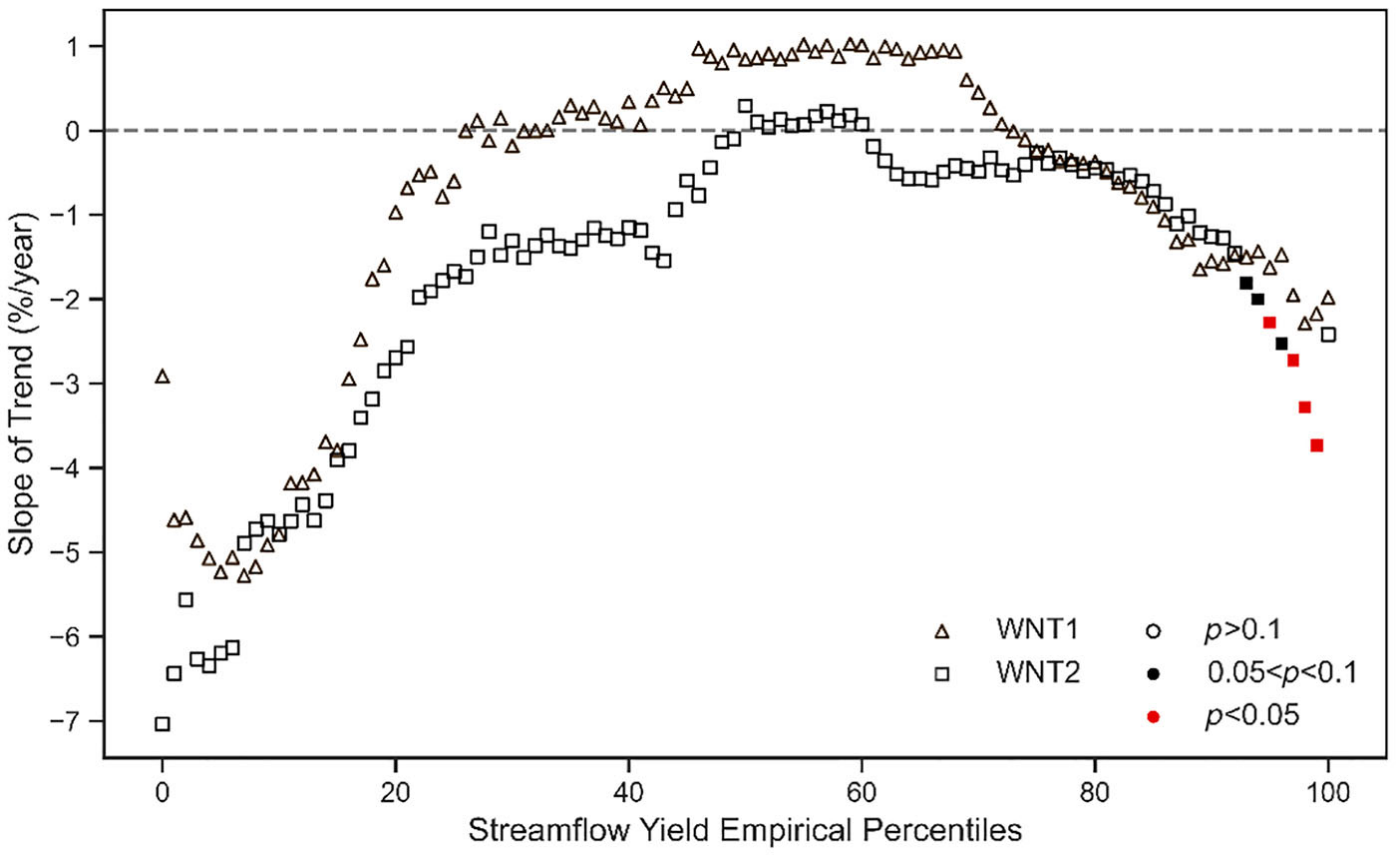
Quantile–Kendall plot showing the slope of trends (from 1995 to 2024) in annual streamflow yield at the upstream monitoring site along Walnut Creek (WNT1) and downstream monitoring site along Walnut Creek (WNT2). Values are color‐coded to indicate statistical significance using *p*‐values from their corresponding trend analysis.

While this analysis quantified the overall impact of the NSNWR on downstream flow, we explored the refuge's influence more comprehensively by conducting trend analyses on each empirical percentile of the annual records. In this approach, we identified the 0th through the 100th empirical percentiles from daily water yields within each year (i.e., the annual minimum flow through the annual maximum flow using individual percentiles). A trend analysis (using the Mann–Kendall test and Theil–Sen slope) was then conducted on the log‐transformed water yields for each percentile. For example, the analysis for the 50th percentile identified the median daily water yield values within each year spanning 1995–2024. We then performed the trend test and slope estimate on this log‐transformed time series of median yields, and this process was replicated for all other percentiles. This assessment is consistent with USGS techniques that examine how streamflow has historically changed under the full range of hydrologic conditions within a watershed (Lins & Slack, 1999). The Theil‐Sen slopes were then plotted and color‐coded by statistical significance to create a Quantile–Kendall plot (Choquette et al., 2019), which summarized the hydrologic trends of WNT1 and WNT2 (Figure 5).

### 2.3 Water quality analysis

Along with streamflow, waterborne nitrate (NO_3_^−^, reported as mg/L as nitrogen) and sediment have been consistently measured at WNT1 and WNT2 and form the focus of our water quality analysis. Both nitrate and sediment have long impaired Iowa's surface water (C. S. Jones, Schilling, et al., 2018; K. Lee et al., 1998), with agricultural activities often a dominant source (C. S. Jones & Schilling, 2011; Schilling & Libra, 2000). Because nitrate and sediment have traditionally been associated with agricultural pollution and degraded water quality in Iowa, monitoring these two parameters has remained a priority since the establishment of the NSNWR (Schilling & Thompson, 2000).

Nitrate is the most prevalent form of nitrogen in central Iowa rivers and streams (Anderson & Schilling, 2024), and environmental concerns arise from its contribution to eutrophication (C. S. Jones, Nielsen, et al., 2018) and toxicity as a drinking water contaminant (Mantey et al., 2025). Nitrate measurements presented in this study comprise both nitrate and nitrite. Because nitrite is highly unstable in Iowa surface waters and often present at low levels (<0.5 mg/L), it is common practice to group these analytes and label them as nitrate (C. S. Jones, Schilling, et al., 2018). Nitrate monitoring at both sites has primarily been conducted via grab sampling at weekly and biweekly intervals.

Sediment has been measured in Walnut Creek using the suspended sediment concentration (SSC) parameter, which includes all suspended particles present in a water column. Sediment pollution remains a concern in Iowa due to its interference with hydraulic operations (Anderson & Schilling, 2025b), degradation of ecosystems (Loperfido et al., 2010), and propensity to carry waterborne pollutants, such as phosphorus (Tomer et al., 2007). Due to an abundance of higher order, more sinuous channels, sediment levels in the lower portions of the Walnut Creek basin are generally larger than those found above WNT1 (Schilling et al., 2011). From 1995 to 2005 (during IDNR sampling), SSC was measured daily, but sampling shifted to weekly when monitoring transitioned to the ARS. Further information on the specific collection and analytical methods used to quantify nitrate and SSC is provided in the Supporting Information.

To evaluate temporal changes in water quality, we estimated annual loads and concentrations of nitrate and SSC using the Weighted Regression on Time, Discharge, and Season with Kalman filtering (WRTDSK) modeling framework. WRTDSK is a widely used statistical method that identifies relationships between water quality measurements, discharge, seasonality, historical trends, and temporal proximity to measured values to produce daily estimates of analyte concentration (Hirsch et al., 2010; Zhang & Hirsch, 2019). WRTDSK has proven successful in the multidecadal modeling of both nitrate (Markus et al., 2014) and sediment in agricultural basins (Chanat et al., 2016; C. J. Lee et al., 2019).

**FIGURE 6:**
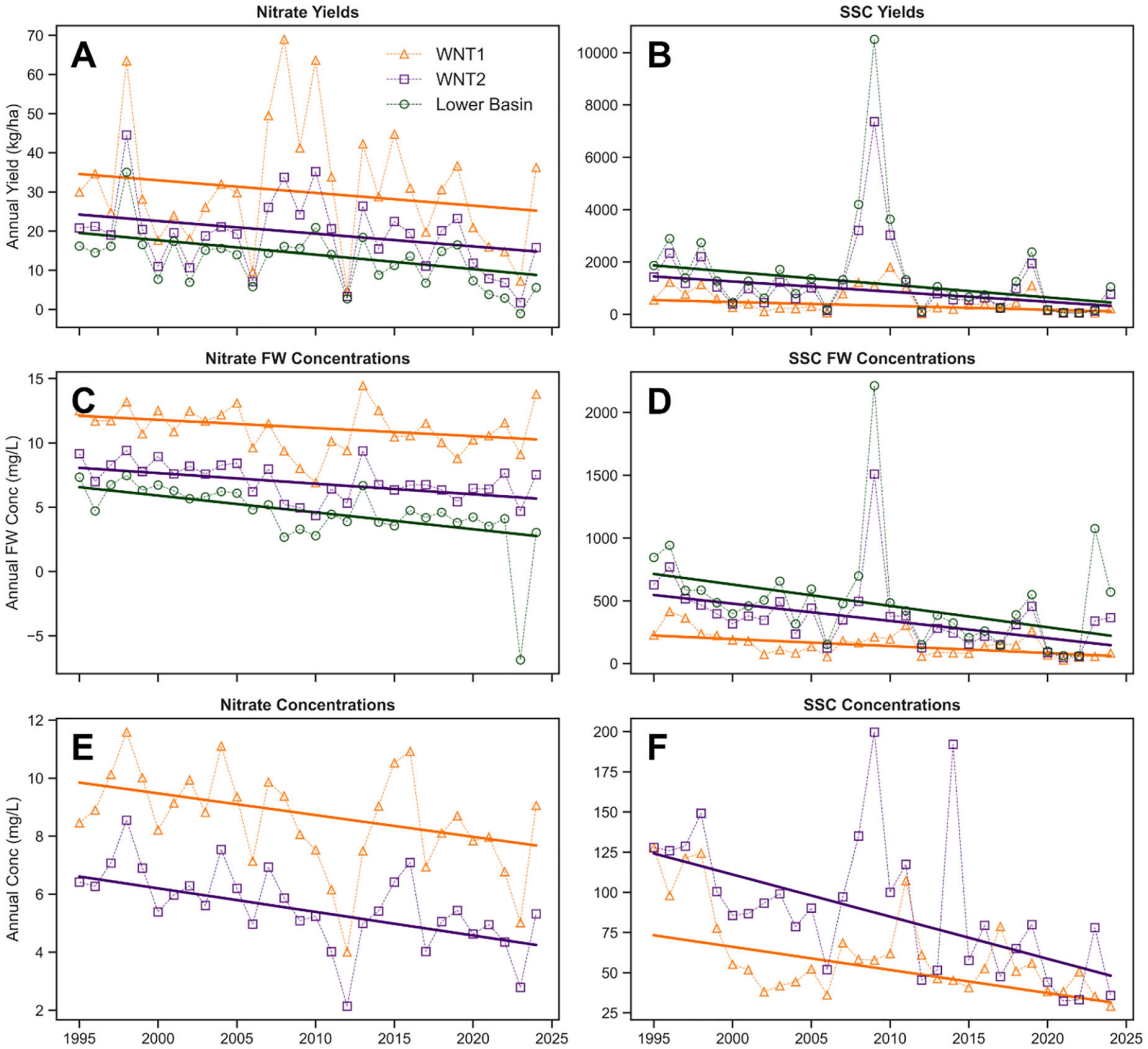
Time series of estimated annual nitrate and suspended sediment concentration (SSC) parameters in Walnut Creek from 1995 to 2024: (A) nitrate yields, (B) SSC yields, (C) nitrate flow‐weighted concentrations, (D) SSC flow‐weighted concentrations, (E) nitrate mean concentrations, and (F) SSC mean concentrations. The solid lines represent monotonic trends. FW conc, flow‐weighted concentrations; WNT1, upstream monitoring site along Walnut Creek; WNT2, downstream monitoring site along Walnut Creek.

**FIGURE 7:**
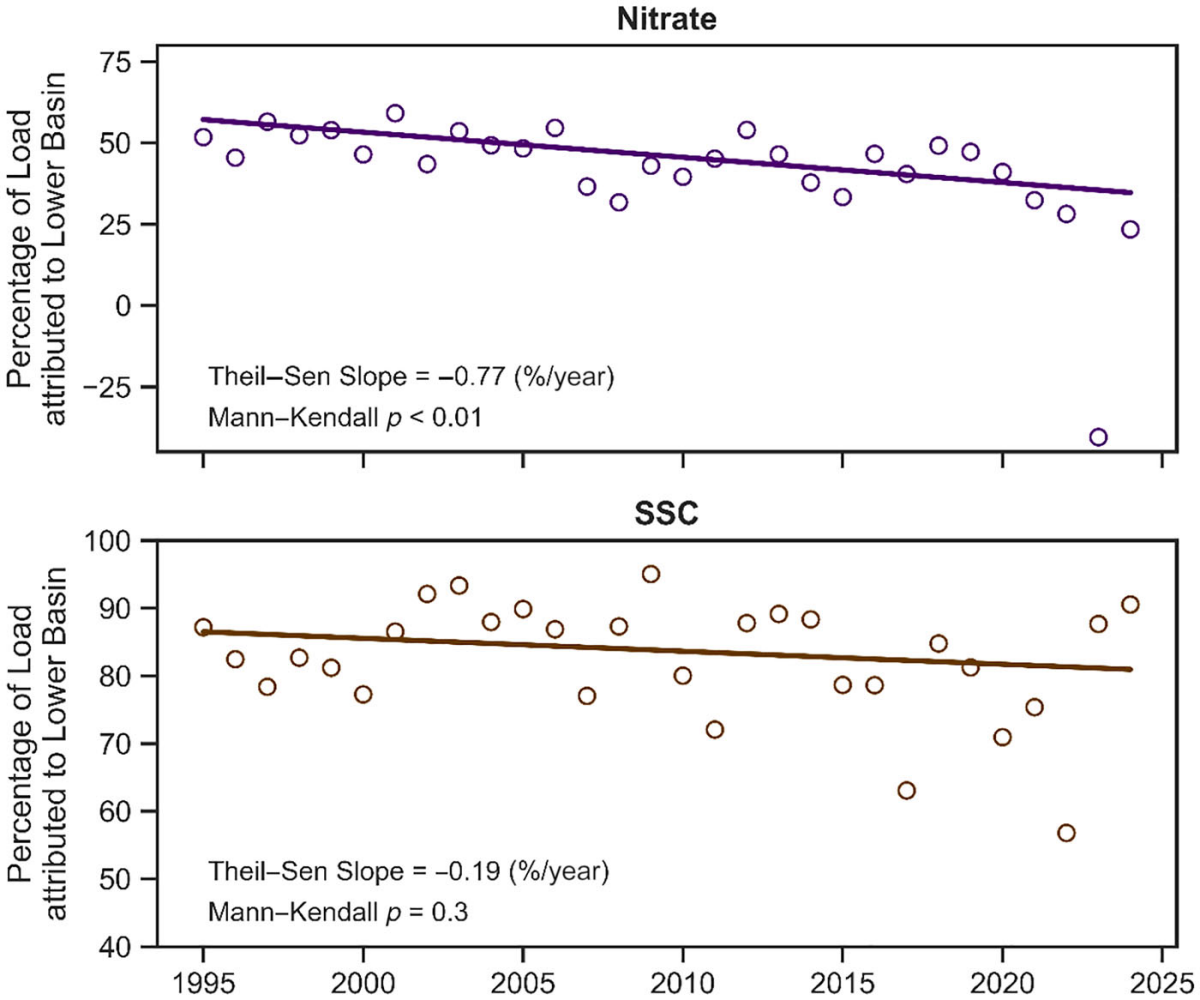
Percentage of annual loads for nitrate (top) and suspended sediment concentration (SSC, bottom) attributed to the Lower Basin. The solid lines represent monotonic trends, with test results provided in the figure text.

Four WRTDSK models were constructed using historical water quality and streamflow datasets; that is, models were created for nitrate and SSC at the WNT1 and WNT2 sites, thereby estimating daily concentration values at each location. These daily estimates were then aggregated annually to produce three metrics: (1) yields (i.e., loads divided by the site's tributary area), (2) flow‐weighted concentrations (FW conc), and (3) mean concentrations (Figure 6). Additionally, yields and FW conc were calculated for the Lower Basin. Much like the streamflow‐based analysis, Mann–Kendall trend tests were conducted on each 30‐year time series (Table 2). We also calculated the percentage of the load attributable to each (Figure 7) and conducted a comparable trend analysis. The Supporting Information contains further materials associated with the water quality analysis, including the datasets used to construct the WRTDSK models, model diagnostics and performance metrics, and estimated annual water quality values.

**TABLE 2:** Results of trend analyses spanning 1995–2024.

	Nitrate slopes			SSC slopes		
Site	Yield (kg/ha/year)	FW conc (mg/L/year)	Mean concentration (mg/L/year)	Yield (kg/ha/year)	FW conc (mg/L/year)	Mean concentration (mg/L/year)
WNT1	−0.323	−0.063	−0.075^*^	−14.8^*^	−5.58^**^	−1.44^**^
WNT2	−0.325	−0.082^**^	−0.081^***^	−38.8^**^	−13.8^***^	−2.62^***^
Lower Basin	−0.370^**^	−0.131^***^		−48.8^**^	−17.0^**^	

*Note*: Values are the slopes of monotonic trends describing annual nitrate and suspended sediment concentration (SSC) parameters (yield, flow‐weighted concentration, and average concentration).

Abbreviations: FW conc, flow‐weighted concentration; WNT1, upstream monitoring site along Walnut Creek; WNT2, downstream monitoring site along Walnut Creek.

^*^, ^**^, and ^***^ denote significance at the 0.05, 0.01, and 0.001 probabilities levels, respectively.

## 3 RESULTS AND DISCUSSION

### 3.1 Hydrology

Annual water yields (1995–2024) ranged from 50 to 921 mm (WNT1), 37 to 807 mm (WNT2), and 13 to 751 mm (Lower Basin), with means of 292, 273, and 264 mm/year, respectively (Table 1; Figure 3). BFI values were 0.66 (WNT1), 0.61 (WNT2), and 0.58 (Lower Basin), while flashiness values were 0.40 (WNT1) and 0.48 (WNT2). It should be noted that flashiness cannot be calculated for the Lower Basin directly, as it is derived from daily streamflow measurements at a given site rather than transposed water volumes. These metrics roughly aligned with typical hydrologic conditions found in smaller Iowa basins (Anderson & Schilling, 2025a), and the 30‐year analysis period encompassed several instances of flooding and drought that affected nearby municipalities (Al‐Kaisi et al., 2013; Gilles et al., 2012), with annual rainfall totals ranging from 594 to 1450 mm. No temporal trends were present for annual precipitation (*p* = 0.48).

The trend test revealed a statistically significant decrease in the percentage of water originating from the Lower Basin (*p* = 0.03), with a rate of −0.23%/year (Figure 4). The Quantile–Kendall plot further revealed the nature of the streamflow changes at WNT1 and WNT2 (Figure 5). For WNT1, the largest decreases (∼‐5%/year) occurred near the fifth percentile. Slopes were approximately 0 for the 20th–40th percentiles and mostly positive for the 40th–70th percentiles. The slopes then began to decline again, culminating with values of −2.5%/year for the maximum flows. The slopes for WNT2 followed a similar pattern, with highly negative values at the lower and upper ends of the streamflow distribution while being closer to 0 for the 20th–80th percentiles. However, the slopes at WNT2 were consistently lower than their counterparts at WNT1. The upper tail of the WNT2 distribution (i.e., the 94th percentile and above) contained the only series with statistically significant trends (*p* < 0.1).

These patterns are consistent with hydrologic responses to prairie restoration. Prairie increases infiltration and evapotranspiration and reduces rapid surface runoff, which can lower annual yields and peak discharges (Schilling & Drobney, 2014; Wine & Zou, 2012). Similarly, the lower BFI is indicative of a more natural midwestern landscape, as agricultural drainage practices have elevated the baseflow component of Iowa's rivers and streams (Schilling & Libra, 2003). These same systems often reduce stream flashiness, as tile lines steadily convey water to nearby streams that would otherwise saturate topsoil and pond in‐field (Green et al., 2006). Tile drainage thereby prevents streams from drying out to the same extent as they would in more natural low‐flow conditions, resulting in lower flashiness (Adelsperger et al., 2023). Therefore, this study's hydrologic statistics largely align with the hypothesized influence of tallgrass prairie on streamflow.

The results of the Quantile–Kendall plot are more nuanced and provide insights into how these changes manifest in the daily streamflow records. The fact that slopes were consistently lower across the WNT2 percentiles suggests that the NSNWR is reducing streamflow across the full range of hydrologic conditions at a rate greater than that of the upstream agricultural catchment. Interestingly, the largest discrepancies between WNT1 and WTN2 occurred between the 20th and 70th percentiles and the highest flow values (>95th percentile). This may indicate the prairie exerts the greatest influence during typical flow conditions and wet weather events. The disruption of tile drainage systems, which often continuously discharge, has likely influenced this change under normal flow conditions, while the prairie's ability to sequester water within its vegetation following heavy rainfall may have led to the statistically significant decreases in peak flows. Indeed, the ability of native vegetation to provide upstream distributed storage across a landscape is well documented (Kharel et al., 2016; Potter, 2011; Srivastava et al., 2023) and has likely played a role in the behavior observed at WNT2.

It is important to note that the WNT1 percentiles were not completely stationary—declines were documented at both the lower and upper ends of the distribution. Many factors beyond land use change, such as climate change (Gupta et al., 2015), shifting agronomic practices (Perry et al., 2009), and variations in point source discharges (Galavi et al., 2019), can also contribute to nonstationary streamflow conditions. Therefore, including a paired watershed (i.e., WNT1) as a control was beneficial for isolating the impact of prairie restoration because the paired watershed captures the effects of other components unrelated to land use change that may be affecting streamflow.

### 3.2 Water quality

Mean annual nitrate yields, FW conc, and concentrations were consistently higher at WNT1 (30 kg/ha/year, 11 mg/L/year, and 8.5 mg/L/year). This was followed by the WNT2 site, which contained means of 21 kg/ha/year (yield), 7.1 mg/L/year (FW conc), and 5.6 mg/L/year (concentration), and the Lower Basin, with means of 16 kg/ha/year (yield) and 4.5 mg/L/year (FW conc). Like stream flashiness, mean concentrations cannot be calculated for the Lower Basin, as they are average concentrations estimated at a particular location. Notably, in the drought year 2023, the Lower Basin acted as a nitrate sink, with more nitrate entering it via WNT1 than outflowing at WNT2, resulting in a negative yield and FW conc. These annual nitrate results indicate that significantly higher (∼2X yield) nitrate losses occurred upstream of the NSNWR than within it (Figure 6A).

The trend analyses revealed decreasing nitrate levels across the monitoring period. Over the 30‐year period, nitrate yields declined at rates of −0.323 (WNT1), −0.325 (WNT2), and −0.370 (Lower Basin) kg/ha/year. FW conc fell at rates −0.063 (WNT1), −0.082 (WNT2), and −0.131 (Lower Basin) mg/L/year, and mean annual concentration slopes were −0.075 (WNT1) and −0.081 (WNT2) mg/L/year. The highest degrees of statistical significance (*p* < 0.001) were observed for declines in FW conc in the Lower Basin (Table 2). Although nitrate decreased at each site, a hierarchy was evident, with slopes constantly lower (i.e., more negative) and more statistically significant in the Lower Basin than in WNT1. Additionally, the percentage of nitrate loads attributable to the Lower Basin also fell at a statistically significant rate (*p* < 0.01; Figure 7 [top]). These results demonstrate the pronounced influence the NSNWR has had on reducing nitrate within the Lower Basin.

In contrast to nitrate, SSC values were consistently larger in the Lower Basin (Figure 6B), which contained a mean annual yield of 1870 kg/ha/year and mean FW conc of 503 mg/L/year. Mean annual SSC yields at WNT1 and WNT2 were 546 and 1430 kg/ha/year, respectively, and mean FW conc were 156 and 369 mg/L/year. These larger SSC values in the Lower Basin are consistent with other studies that describe a greater propensity for streambank erosion within this portion of Walnut Creek (Schilling & Drobney, 2014; Schilling et al., 2011; Schilling & Wolter, 2000; van der Burg et al., 2017). Notably, a single high‐flow event in 2009 resulted in disproportionately high sediment transport in the Lower Basin that year.

The SSC trend analysis also produced decreasing trends across the monitoring period. The slopes for yields were −14.8 (WNT1), −38.8 (WNT2), and −48.8 (Lower Basin) kg/ha/year. For FW conc, slopes were −5.58 (WNT1), −13.8 (WNT2), and −17.0 (Lower Basin) mg/L/year, and for mean concentrations, they were −1.44 (WNT1) and −2.62 (WNT2). For the SSC trends, all slopes (apart from the WNT1 yield) proved highly statistically significant (*p* < 0.01; Table 2). A hierarchy was again observed, with SSC declines in the Lower Basin always steeper (i.e., more negative) than those at WNT1. The percentage of the load attributable to the Lower Basin also fell, but here the trend was not statistically significant (*p* = 0.3; Figure 7 [bottom]). Overall, these results suggest the NSNWR has reduced sediment transport within the Lower Basin.

The ability of the NSNWR to reduce both nitrate and sediment is consistent with long‐established ecological principles suggesting that prairies and unaltered riparian corridors foster denitrification (Groffman et al., 1993; Kemp & Dodds, 2002) and lessen surface runoff and streambank erosion (Helmers et al., 2012; Zaimes et al., 2006). Therefore, the documented influence of the NSNWR on nitrate and SSC is consistent with the hypothesized impact on nutrient reduction and erosion control. While this behavior has often been observed at a field scale, our results suggest that these same reductions can be observed at a watershed scale with sufficient upstream land conversion. Critically, the NSNWR consistently produced greater reductions than its counterpart (WNT1), which contained an unaltered agricultural landscape. This occurred across all metrics for both parameters despite their uneven presence throughout Walnut Creek.

### 3.3 Implications for conservation efforts

This study establishes that when sufficient portions of a basin's tributary area are converted from conventional row crop to grassland, downstream decreases in flow rates and nutrient and sediment levels are observable. While water quality improvements resulting from this sort of conversion have repeatedly been documented at the field scale with individual plot studies (Steinke et al., 2009; Stephenson et al., 2024; X. Zhou et al., 2014), demonstrating tangible benefits at the watershed scale has proven more elusive. Most attempts to describe the downstream effects of large‐scale landscape alterations have relied on models that simulate pollutant fate and transport (Jha et al., 2007) rather than water quality observations (Erol & Randhir, 2013). Several challenges often arise when upscaling the results of plot experiments to an entire basin. First, basin‐wide studies necessitate that considerably more cropland be converted than is typically done in small catchments, often requiring substantial labor and financial resources (Long et al., 2021). Second, assessments at larger spatial scales typically introduce greater uncertainty into experimental parameters, as it is more difficult to document all environmental processes across a watershed than in a single field. Uncertainties surrounding sources and transport pathways of nutrients and sediment can confound the effects of land use conversion (Tong & Chen, 2002). In practice, the combination of these two challenges often results in basin‐scale improvements taking longer to materialize at statistically significant levels than they would at a field scale, where reductions are more immediate (Ni et al., 2021). Walnut Creek is thus a rare example of a watershed with significant landscape restoration and sufficient long‐term monitoring data to conclusively demonstrate the benefits of land use conversion.

The results of our study are consistent with the expected outcomes of converting cropland to tallgrass prairie, in that streamflow, nitrate, and SSC yields all experienced greater declines downstream of NSNWR than in the upstream unaltered landscape. Still, the temporal rates of nutrient and sediment declines associated with conservation are not fully understood (Calijuri et al., 2015). For example, when Schilling and Spooner (2006) explored nitrate reductions in Walnut Creek after the first decade of monitoring, they hypothesized that annual decreases in nitrate concentrations would accelerate following marginal declines observed over the first 10 years. Likewise, after the second decade of monitoring, Tomer et al. (2019) expected nitrate declines to increase as prairie reconstructions became better established. However, our study suggests that the rate of nitrate decline has remained relatively consistent across 1995–2024 (Figure 6A,C,E), despite minimal prairie restoration taking place after 2005. Interestingly, nitrate declines also took place upstream of the NSNWR—albeit to a lesser extent than those within the Lower Basin. This may be due to improved nitrogen application in upstream croplands (i.e., applications of synthetic fertilizer and manure that better follow agronomically prescribed rates), since nitrate dilution seems unlikely, given the lack of rainfall and streamflow trends across our study period. We are unaware of any appreciable conservation adoption or land use changes upstream of NSNWR, so these declines likely result from a combination of enhanced nitrogen use efficiency and the timing of several years with low water yields near the study period's conclusion (2021–2023).

While nitrate contributions from the NSNWR portion of the basin undoubtedly fell (Figure 7 [top]; *p* < 0.01), the rate of decline has not substantially increased or plateaued throughout the 30‐year analysis period. This may be due to travel times associated with groundwater flow. Although Walnut Creek's mean groundwater residence time has been estimated at approximately 10 years, a wide range of travel times has been noted throughout the basin, with some exceeding 50 years (Schilling & Wolter, 2007). This variability in travel times may be dispersing the delivery of legacy nitrate to the monitoring station at WNT2. In practice, this could smooth nitrate attenuation in Walnut Creek and lead to more gradual long‐term declines. A prominent mechanism for nitrate reduction following cropland conversion to grassland is denitrification in hypoxic shallow groundwater (Smith et al., 2013). This process is enhanced by the presence of vegetation and the removal of tile, both of which increase infiltration (Schipper et al., 2004). Additionally, the decades of row crop cultivation have likely built up substantial amounts of legacy nitrogen in NSNWR's soil, which can persist in downstream nutrient loads for many years (Van Meter et al., 2016). Therefore, nitrate sources within NSNWR have likely not been extinguished, resulting in the ongoing, relatively consistent declines noted in our study.

SSC yields also fell at relatively consistent rates, but the situation surrounding SSC is more complex. While SSC loads fell, its decline within the NSNWR portion of Walnut Creek was not statistically significant (Figure 7 [bottom]; *p* = 0.3). This may result from the specific type of erosion control most associated with cropland conversion (i.e., sheet and rill erosion) not being a major contributor to sediment within Walnut Creek. Schilling et al. (2011) found that following the establishment of NSNWR, SSC yields in Walnut Creek were not significantly lower than in a paired agricultural watershed, and this could mainly be explained by streambank erosion being the dominant sediment delivery mechanism. It has been shown that in small upland catchments where prairie has completely replaced row crop cultivation, sediment export from prairie catchments is significantly less than in 100% cropped catchments (Helmers et al., 2012; Schilling & Drobney, 2014). However, improvements in catchments may not be sufficient to overcome the influence of streambank erosion on sediment export at the watershed scale. Studies in Walnut Creek have shown that the annual contribution of streambank erosion to total sediment export ranged from 6% to 75%, varying with discharge frequency and magnitude (Palmer et al., 2014). Hence, we suggest that the SSC declines we observed in Walnut Creek may not stem entirely from on‐field sediment retention but also from decreases in streambank erosion. Indeed, the reduced streamflows noted in our hydrologic analysis may have reduced streambank erosion, as lower flow rates and improved infiltration have been noted to curtail riverine meandering by dissipating stream power (Vietz et al., 2018; Zaimes & Schultz, 2015).

Finally, our study highlights the advantages of conducting long‐term (i.e., multidecade) paired monitoring to identify the impact of land use conversion on downstream water quality. In addition to the challenges associated with watershed‐scale assessments, water quality trend analyses are complicated by the inherent hydrologic variability that affects pollutant transport. Because annual nitrate and sediment levels are heavily related to the amount of rain each year, variations in annual precipitation can obscure the effects of a conversion practice. The best remedy for this hydrologic variability is lengthening the monitoring and analysis timeframe (Hirsch et al., 1991), and researchers have noted that it often takes decades for the impacts of upstream conservation throughout a watershed to be detectable (Schreiber et al., 2022). Studies that span fewer than 5 years are likely to be insufficient for identifying basin‐scale behavior (Hirsch et al., 2015). Furthermore, attributing the nitrate and SSC declines to the influence of the NSNWR would not have been possible without paired upstreaming monitoring. Since many factors can influence riverine nutrient and sediment, comparing downstream loads and concentrations to their upstream counterparts allowed us to isolate the specific impact of the refuge's land use change. Analyses that focus on a single watershed monitoring point risk being influenced by factors outside the conservation practice they aim to explore (Snelder et al., 2021).

Large‐scale land use conversion efforts in southern Iowa would likely yield results similar to those documented in this study. There are numerous basins with geologic, topographic, climatic, and land use characteristics comparable to those of Walnut Creek prior to the establishment of NSNWR (Prior, 1991; Rundhaug et al., 2018). While we reported statistically significant reductions of nitrate and SSC loads and concentrations, our results are difficult to generalize or apply to other watersheds, since the values reported in this study stem from the specific location, extent, and timing of prairie reconstruction in Walnut Creek, along with hydrologic conditions during 1995–2024. Other watershed management projects should carefully consider site‐specific criteria when setting water quality improvement goals associated with land use conversion.

## 4 CONCLUSIONS

Based on our analysis, we conclude that the construction of the NSNWR has improved water quality in Walnut Creek by reducing nitrate and SSC. Walnut Creek is a rare example of a watershed that has experienced substantial enough land use change (through prairie reconstruction) to demonstrate tangible water quality improvements. Even in this “best‐case” scenario, documenting the pollutant reduction associated with prairie reconstruction was not straightforward. It was only made possible through long‐term (i.e., multidecadal) monitoring of both the NSNWR and a paired watershed upstream that did not undergo land use change. While future prairie reconstructions will almost certainly improve water quality, predicting the rates at which these improvements will occur is challenging, because local spatiotemporal factors highly influence pollutant transport.

## AUTHOR CONTRIBUTIONS

**Elliot S. Anderson**: Conceptualization; formal analysis; methodology; visualization; writing—original draft. **Keith E. Schilling**: Conceptualization; investigation; methodology; validation; visualization; writing—review and editing. **Kevin J. Cole**: Data curation; methodology; validation; writing—review and editing. **Kenneth M. Wacha**: Data curation; methodology; validation; writing—review and editing.

## CONFLICT OF INTEREST STATEMENT

The authors declare no conflicts of interest.

## Supporting information



The Supporting Information details the analytical and collection methods used to obtain the streamflow, nitrate, and SSC data utilized in this study. Each of these datasets has also been included. Additionally, the Supporting Information contains the error metrics and residual plots from the WRTDSK models used to estimate daily nitrate and SSC concentrations. All annual values (i.e., annual yields, flow‐weighted concentrations, and average concentrations) have also been included.
