# Supplementary material for: Watershed‐scale effects of tallgrass prairie reconstruction: 30‐Year trends in streamflow, nitrate, and sediment in Walnut Creek, Iowa
Source: J Environ Qual. 2026 Apr 5;55(2):e70174. doi: 10.1002/jeq2.70174 (PMC13051032; doi:10.1002/jeq2.70174)
Supplement: Supplementary file 1 — The Supporting Information details the analytical and collection methods used to obtain the streamflow, nitrate, and SSC data utilized in this study. Each of these datasets has also been included. Additionally, the Supporting Information contains the error metrics and residual plots from the WRTDSK models used to estimate daily nitrate and SSC concentrations. All annual values (i.e., annual yields, flow‐weighted concentrations, and average concentrations) have also been included. [file JEQ2-55-0-s001.zip › supplemental/analytical methods/ars_autosampler_publication.pdf]

Automated Storm Water Sampling on Small Watersheds

R. D. Harmel, K. W. King, R. M. Slade

Abstract. *Few guidelines are currently available to assist in designing appropriate automated storm water sampling strategies for small watersheds. Therefore, guidance is needed to develop strategies that achieve an appropriate balance between accurate characterization of storm water quality and loads and limitations of budget, equipment, and personnel. In this article, we explore the important sampling strategy components (minimum flow threshold, sampling interval, and discrete versus composite sampling) and project-specific considerations (sampling goal, sampling and analysis resources, and watershed characteristics) based on personal experiences and pertinent field and analytical studies. These components and considerations are important in achieving the balance between sampling goals and limitations because they determine how and when samples are taken and the potential sampling error. Several general recommendations are made, including: setting low minimum flow thresholds, using flow-interval or variable time-interval sampling, and using composite sampling to limit the number of samples collected. Guidelines are presented to aid in selection of an appropriate sampling strategy based on user's project-specific considerations. Our experiences suggest these recommendations should allow implementation of a successful sampling strategy for most small watershed sampling projects with common sampling goals.*

Keywords. *Storm water sampling, Automated sampling, Nonpoint source pollution, Water quality.*

The realization that nonpoint source (NPS) pollution continues to adversely impact rivers, lakes, and coastal waters (USEPA, 1995, 2000) has forced the water resource community to undertake the difficult task of monitoring storm water quality. The traditional monitoring focus on periodic grab sampling to characterize point source pollution (discharged from specific locations such as factories and waste water treatment plants) must often be supplemented with storm flow monitoring to characterize NPS pollution. NPS pollution is generated when rainfall runs off from diffuse sources such as urban areas, farms, and silvicultural operations. The types of NPS pollution are as varied as the sources, but NPS pollution typically involves nutrients, pathogens, sediment, metals, and pesticides. In water bodies, excessive pollutants can degrade aquatic ecosystem health, increase water treatment costs, and diminish recreational and aesthetic value.

To sample storm water for NPS pollutants, sampling projects on small watersheds typically utilize automated sampling equipment. Most commercially available automated samplers contain similar components, which include: programmable operation and memory, water level recorder, sample collection pump, and sample bottles. We currently use ISCO samplers (ISCO, Inc., Lincoln, Nebr.) in our studies, but automated samplers are also available from American Sigma, Inc. (Loveland, Colo.), Global Water Instrumentation (Gold River, Calif.), Intermountain Environmental (Logan, Utah), and other companies. Typical sampler operation involves: 1) setting a minimum flow threshold to start and finish sampling (typically a flow depth, possibly including a rainfall depth per specified time); 2) setting a time or flow interval on which to collect samples after the sampler is triggered; and 3) determining whether to take discrete or composite samples.

Storm sampling projects for small watersheds typically use automated sampling equipment because manual sample collection is especially difficult because of relatively short storm runoff durations. Also, manual sampling requires personnel prepared to travel to often remote sampling sites and work in dangerous locations under adverse weather conditions with little advance warning. In sampling projects on larger streams and rivers, however, manual sampling is commonly used to obtain multiple samples across the stream cross-section and adequately represent pollutant concentrations. Wells et al. (1990) gives extensive guidance on manual field measurements in terms of sample collection techniques and quality control. Several recent studies including Stone et al. (2000), Izuno et al. (1998), and Robertson and Roerish (1999) have evaluated the impact of various sampling strategy components. These studies generally address differences in pollutant concentration and load estimates resulting from using different sampling strategy components.

This article focuses on automated strategies for sampling "edge of field" and small stream conditions in homogeneous watersheds or in watersheds with a limited number of

Article was submitted for review in October 2002; approved for publication by the Soil & Water Division of ASAE in June 2003.

Mention of trade names or commercial products is solely for the purpose of providing specific information and does not imply recommendation or endorsement by the United States Department of Agriculture.

The authors are **R. Daren Harmel, ASAE Member Engineer,** Agricultural Engineer, USDA-ARS, Grassland, Soil and Water Research Laboratory, Temple Texas; **Kevin W. King, ASAE Member Engineer,** Agricultural Engineer, USDA-ARS, Soil Drainage Research Unit, Columbus, Ohio; and **Raymond M. Slade,** Hydrologist, Texas District Surface-Water Specialist (retired), United States Geological Survey, Austin, Texas. **Corresponding author:** R. Daren Harmel, USDA-ARS, 808 East Blackland Road, Temple, TX 76502; phone: 254-770-6521; fax: 254-770-6561; e-mail: dharmel@spa.ars.usda.gov.

subwatershed contributions, collectively referred to as small watershed studies. For these strategies, it is assumed that water quality can be adequately sampled at a single intake point in the stream, which is generally valid for small streams because of well-mixed conditions. Our specific objectives are: 1) to share information and experiences on sampling strategy components and other considerations gained in field and analytical studies, and 2) to make general recommendations for developing automated storm sampling strategies for small watersheds based on these results and experiences.

Sampling Strategy Components

As stated above, automated sampler operation typically involves setting a minimum flow threshold, setting an interval on which to collect samples, and determining whether to take discrete or composite samples. These sampling strategy components affect the timing, frequency and number of samples taken, which in turn affect sampling and analysis costs and data quality (Novotny and Olem, 1994). Therefore, it is important to understand sampling component impacts and interactions when developing a sampling strategy. With this understanding, sampling strategies can be developed that satisfy project goals within budget, personnel, and laboratory constraints.

Minimum Flow Thresholds

Developing a storm water sampling strategy requires selecting a minimum flow depth threshold above which to trigger sampling. When flow depth exceeds this minimum level, sampling begins and continues as long as the flow remains above this level; therefore, the minimum flow threshold directly affects the number of samples taken (fig. 1). With time- and flow-interval sampling, a high minimum flow threshold reduces the number of samples and increases the difference between the measured and true pollutant flux. In contrast, a low minimum flow threshold increases the number of samples taken, which in turn may exceed sampler capability and lab analysis resources.

Results from Harmel et al. (2002) suggest that substantial error is introduced as minimum flow thresholds are increased. Therefore, we recommend that minimum flow thresholds be set such that even small storms with small increases in flow depth are sampled. On four small watersheds ranging from 6 to 67 ha studied by Harmel et al. (2002), minimum flow thresholds of 0.001 to 0.04 m^3/s are recommended (thresholds are indicated as flow rates for consistency because flow depth varies significantly as a function of stream or hydraulic structure geometry). To prevent pump malfunction, it is necessary to insure that the sample intake will be submerged at the minimum flow threshold. If reducing the number of samples collected is necessary, minimum flow thresholds should be increased only after careful consideration of the consequences.

Automated samplers generally have the option to take a sample when flow rises and/or falls past the minimum flow threshold (i.e., as sampling is initiated and completed). We recommend not sampling as flow passes the minimum flow threshold because flow can fluctuate back and forth across the threshold thus taking excessive samples.

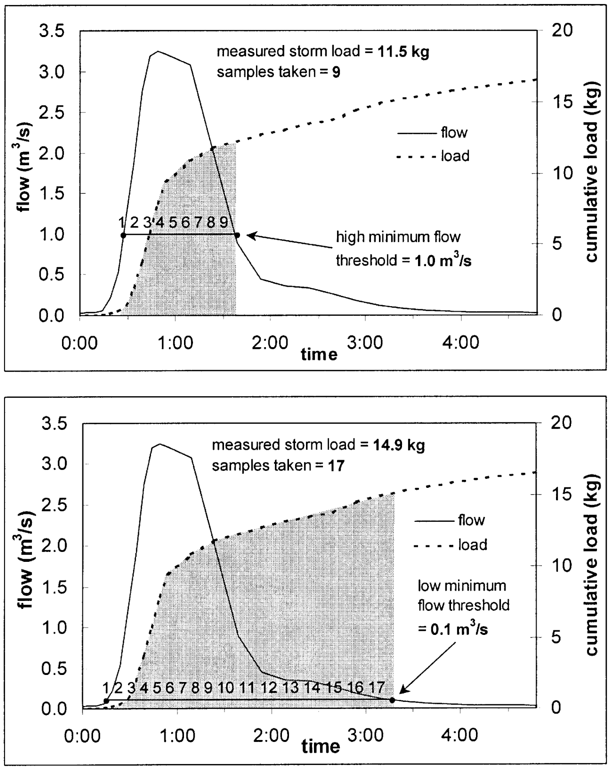

Figure 1. Measured loads for two minimum flow thresholds (examples for 10-min time-interval sampling - shaded areas represent measured portion of total load, samples indicated by numbers).

Sampling Based on Time or Flow Intervals

Another important consideration is setting the time or flow interval on which to sample once the flow level exceeds the minimum flow threshold. If samples are taken based on a specified duration (such as every 30 min), sampling is referred to as time-interval sampling (fig. 2). Similarly, if samples are taken based on a specified flow volume (such as every 2000-m^3 or 2.5-mm volumetric depth), sampling is referred to as flow-interval sampling (fig. 2). Typically, samples are taken on a constant time or flow interval. Samples can also be taken on variable time or flow intervals, especially if data on hydrologic characteristics are available on which to base intervals.

Several studies have addressed the differences between time- and flow-interval sampling. Based on these studies, flow-interval sampling generally best represents storm loads because a greater proportion of samples are taken at higher flow rates (fig. 2); however, time-interval strategies are more simple and economical when only concentrations are needed (Shih et al., 1994; McFarland and Hauck, 2001; Rekolainen et al., 1991; Richards and Holloway, 1987; Claridge, 1974; King and Harmel, 2003; Izuno et al., 1998; Miller et al., 2000).

Time-interval sampling is a simple and reliable procedure since accurate time intervals are easy to measure and clock failures are rare. However, if small time intervals are used, frequent sampling will produce a large number of samples and will limit the length of storm duration that can be sampled within sampler capacity. With time-interval sampling, flow measurements are needed for pollutant load calculations.

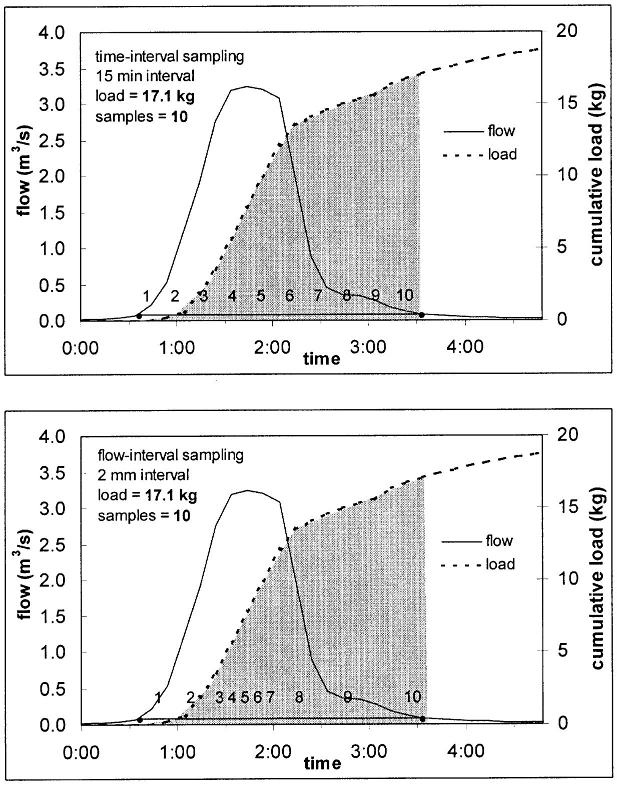

Figure 2. Example storm illustrating time- and flow-interval sampling (shaded areas represent measured portion of total load, samples indicated by numbers).

A major advantage of flow-interval sampling is more frequent sampling during high flows; however, flow-interval sampling requires continuous flow monitoring to determine sampling intervals, even if only concentrations are desired. Concentrations from individual flow-interval samples can be easily averaged to produce a flow-weighted mean concentration. This concentration is commonly referred to as the Event Mean Concentration (EMC), which by definition is the arithmetic mean of individual sample concentrations collected on equal discharge intervals. The EMC multiplied by the total flow volume represents the storm load.

To best represent true loads, short time or small flow interval samples are needed. Statistical sampling theory indicates that the smaller the sampling interval (the more samples taken), the better actual population characteristics are estimated (Haan, 1977). This theory is supported in storm monitoring by several studies including King and Harmel (2003), Miller et al. (2000), and Richards and Holloway (1987). However, smaller intervals increase the number of samples taken, which limit the storm duration or magnitude that can be completely sampled because of sampler bottle capacity. Therefore, a compromise between sampling interval and sample numbers must be reached.

Increasing the sampling interval introduces less error (relative to the reduction in samples to be analyzed) than raising minimum flow thresholds (Harmel et al., 2002). King and Harmel (2003) reported the number of samples taken per storm for various sampling strategies for 300 storms on watersheds from 0.1 to 6294 ha. In that study, time-interval discrete strategies from 5 to 360 min and flow-interval discrete strategies from 2.5 to 15.0 mm volumetric depth along with composite strategies of three and six samples per bottle were examined (discrete and composite sampling are discussed below). Table 1 presents data on the number of samples taken for the discrete strategies for 190 storms in the watersheds less than 1000 ha. The number of samples taken for composite strategies can be determined by dividing the number of discrete samples by the number composited (three and six). Similarly, the number of samples for a flow interval of 1 mm was calculated as 2.5 times the number for the 2.5-mm interval.

When setting a sampling interval, it is important to realize that sampling equipment is limited in terms of time required to collect a sample. Automated samplers purge the sample line before taking a sample, collect a sample, and then clear the sample line again. This process can take from 1 to 2 min depending on the length of the sample tube and sample volume. For time-interval sampling, this does not pose a problem because time-intervals greater then 5 min are generally required to produce a reasonable number of samples. For flow-interval sampling, however, this equipment limitation may be a problem in rare cases. For the 300 storms analyzed in King and Harmel (2003), five storms had peak flow rates greater then 1-mm/min volumetric depth, which required more than 1 sample/min and exceeded sampler capabilities.

Discrete and Composite Sampling

Another issue to consider when developing a storm sampling strategy is whether to take discrete samples (collection of one sample per bottle) or to composite samples (collection of more than one sample per bottle). Composite sampling decreases sample numbers and permits longer duration and larger magnitude events to be sampled (McFarland and Hauck, 2001). Table 2 illustrates the increases in storm duration and magnitude that can be achieved with

Table 1. The number of samples taken for each time- and flow-interval discrete sampling strategy for storms on watersheds less then 1000 ha, adapted from King and Harmel (2003).

Sampling Strategy	Number of Samples Taken				
Time-Interval				Percentiles	
Discrete (min)	Range	Mean	Median	10th	90th
5	8 - 1237	234	164	72	475
10	4 - 619	117	82	36	238
15	3 - 413	78	55	24	159
30	2 - 207	39	28	12	80
60	0 - 104	20	14	6	40
120	0 - 52	10	7	3	20
180	0 - 35	6	5	2	14
300	0 - 21	4	3	2	8
360	0 - 18	3	3	0	7
Flow-Interval				Percentiles	
Discrete (mm)	Range	Mean	Median	10th	90th
1.0	0 - 132	30	25	0	65
2.5	0 - 53	12	10	0	26
5.0	0 - 26	6	5	0	13
7.5	0 - 17	3	3	0	8
10.0	0 - 13	2	2	0	6
12.5	0 - 10	2	2	0	5
15.0	0 - 8	1	1	0	4

Table 2. Storm duration and volume capabilities for various discrete and composite strategies (assuming a 24-sample bottle limitation).

Time Interval (min)	Maximum Storm Duration That Can Be Sampled (min)			
	Discrete	2/bottle	4/bottle	6/bottle
5	120	240	480	720
10	240	480	960	1440
15	360	720	1440	2160
30	720	1440	2880	4320
60	1440	2880	5760	8640
120	2880	5760	11520	17280
180	4320	8640	17280	25920
300	7200	14400	28800	43200
360	8640	17280	34560	51840

Flow Interval (mm)	Maximum Storm Volume That Can Be Sampled (mm)			
	Discrete	2/bottle	4/bottle	6/bottle
1.0	24	48	96	144
2.5	60	120	240	360
5.0	120	240	480	720
7.5	180	360	720	1080
10.0	240	480	960	1440
12.5	300	600	1200	1800
15.0	360	720	1440	2160

composite sampling (assumes a 24-sample bottle limitation). The disadvantages of compositing are the decrease in pollutant distribution data within the storm and the possible increase in load error estimates, especially in time-interval schemes (King and Harmel, 2003; Miller et al., 2000). Composite sampling does, however, introduce less error (relative to the reduction in samples to be analyzed) than raising minimum flow thresholds and can also introduce less error than increasing sampling intervals (Harmel et al., 2002; Miller et al., 2000). This important compromise between sample numbers and measurement uncertainty should be considered when deciding between discrete versus composite sampling. If a decision is made to composite samples, flow-interval sampling is recommended. Though flow-interval sampling is more complicated than time-interval sampling, concentration and load results from flow-interval composite sampling are generally more useful. Even with composite flow-interval sampling, concentrations can be easily averaged and multiplied by the corresponding flow volume to determine loads.

Additional Considerations

These sampling strategy components may need to be adjusted based on monitoring goals and resources and on watershed hydrologic characteristics. Therefore, in order to develop appropriate sampling strategies, it is important to understand the interaction between strategy components and project-specific considerations.

Sampling Project Goal

The specific objectives of storm water sampling projects vary based on project needs, but common goals include: 1) comparing water quality impacts of various land management activities, 2) evaluating water quality improvement following implementation of best management practices, 3) determining annual pollutant fluxes for Total Maximum Daily Load (TMDL) projects, 4) providing input for watershed modeling, 5) identifying temporal changes or trends in water quality, 6) identifying existing or emerging water quality problems, 7) testing for regulatory compliance, and 8) locating high load "hot spot" areas (Novotny and Olem, 1994; Tate et al., 1999; Robertson and Roerish, 1999; McFarland and Hauck, 2001). Since overall goals vary greatly from project to project, careful consideration should be given to specific sampling goals and needs. Project goals do affect decisions on setting the parameters discussed previously because decisions on minimum flow threshold, time- versus flow-interval sampling, and discrete versus composite sample collection affect sampling numbers, timing, frequency, costs, and sampling error. Therefore, all of these considerations together affect the relative success of automated sampling projects.

One sampling goal consideration is whether pollutant concentration data are adequate or if pollutant load data are needed. In some projects such as regulatory testing, concentration data may be adequate to determine compliance. In this case, equipment and effort to measure flow rate may be unnecessary. However, if flow-weighted concentrations or EMC's are required, flow measurement equipment is needed. If maximum storm concentrations are needed, discrete samples must be taken and analyzed. For projects that require pollutant load determination, for example a nutrient TMDL project for a water supply reservoir, both concentration and flow data are needed. In these projects, flow-monitoring equipment is needed whether time- or flow-interval sampling is used. In certain projects, it is necessary to know how pollutant concentrations change during storm events. Research studies on mechanisms of rainfall and pollutant interaction and studies involving acute toxicity to ammonia or pesticides are examples. In these studies, discrete samples are needed to track concentration changes throughout the storm. If knowledge of within storm variation is not needed, then composite sampling may be adequate. If samples are composited, flow-interval sampling is recommended because EMC's and storm load values are easily calculated from flow-weighted results.

If measurement of within-storm concentration change is unnecessary, many small volume flow-interval samples can be composited into a single collection bottle. With 100- to 200-mL sample volumes (the smallest samples we recommend), 75 to 150 flow-interval samples can be composited into a large sample bottle (16-L capacity), which allows complete sampling of large runoff events. The concentration from that composite sample is the EMC and when multiplied by the runoff volume represents the storm load. Shih et al. (1994) presents this single-bottle flow-interval strategy as a viable sampling option to control costs, and our experience supports this result. A similar strategy with time-interval composite sampling can be used to collect a time-averaged concentration.

Sampling and Analysis Resources

Sampling strategies must fall within project budget limitations. In order to meet reasonable sampling expectations with the project goal in mind, sampling plans or proposals should specify a maximum number of storms that will be sampled or a maximum number of samples that will be collected. Service and maintenance of automated sam-

pling equipment is labor intensive and expensive, and cost considerations often limit the number of samples that can be collected and analyzed (Shih et al., 1994; Robertson and Roerish, 1999; Dissmeyer, 1994). Another consideration in developing a sampling strategy is the number of samples that can be collected and analyzed by a laboratory in a reasonable time frame (Novotny and Olem, 1994). Since a large portion of the cost of a monitoring program is directly related to the number of samples, determination of a proper minimum flow threshold and sample frequency is important in achieving objectives within budget limitations. A high minimum flow threshold and/or low frequency sampling may bypass important information and lengthen project duration. However, funding limitations may inhibit a low minimum flow threshold and/or high sampling frequency.

Another important budget factor is whether to use a flow control structure such as a flume or weir in which to measure flow. Purchase and installation of these structures is quite expensive, but use is recommended if the budget allows. Installation of a hydraulic control structure with a known stage-discharge relationship eliminates the need to develop a stage-discharge relationship in the field. Stage-discharge relationships are important because they relate flow depth, which is relatively easy to measure, to flow rate. Developing a stage-discharge relationship requires measuring flow depths, areas, and velocities for a range of flow depths during runoff events. This process can require substantial time, which in a short study may not be feasible. With minimal maintenance, structures can provide reliable flow data for a number of years. The presence of a hydraulic control structure also improves the accuracy of flow measurement and therefore flow-interval sampling. In contrast, for a sampling station in a natural channel subject to morphological shifts, periodic verification and adjustment of the stage-discharge relationship is necessary.

If installation of a hydraulic structure is not feasible, location of the sampling site at or near an established gaging station with available data is a preferred alternative. Other preferred sampling site locations are culverts or concrete channels. These locations provide a consistent stage-discharge relationship that can be adapted from general hydraulic equations and used for the site. Location of a sampling site in a stream where little or no data are available creates considerable difficulty in making reliable flow measurement.

Maintenance of automated sampling equipment is an expensive and time-consuming task, but it must be done to limit malfunctions during storm events. Duplicate equipment serving as a backup or substitute for malfunctioning equipment represents another wise investment. We recommend weekly or every other week maintenance visits to check power sources, pumps, sample tubes, sample intakes, and dessicant levels. These routine maintenance checks will also limit malfunctions during storms. We also recommend frequent flow level calibration to ensure accurate flow measurement and frequent data retrieval to avoid data loss due to power failure or other malfunction. It is also recommended to return to all sampling sites as soon as possible after storm events to collect samples, check sampler function, and make necessary repairs.

Watershed Hydrologic Characteristics

Hydrologic characteristics of the watershed should be considered when developing a small watershed sampling strategy. Hydrologic characteristics such as runoff volume, rainfall depth, storm duration, storm intensity, and return frequencies can be especially helpful in developing sampling strategies. Therefore, it is important make use of available watershed data in strategy development and site determination.

Runoff duration information is especially helpful in developing watershed-specific variable time-interval strategies. For example, to provide adequate resolution in short duration events and adequate sampling capacity for longer events, variable time-interval strategies can be developed with small intervals during the initial portion of the storm (rising portion of the hydrograph) and increasing intervals as the storm progresses. For flow-interval strategies, historical annual runoff volumes can be used to estimate the range and average number of samples per year. For example, if a watershed has an average annual runoff of 300 mm, then a discrete flow-interval strategy based on 2-mm intervals will produce an average of 150 samples per year. Similarly, the range of annual runoff values would give an expected range of sample numbers. Even if annual runoff data are not available, regional annual rainfall data and runoff estimates can give an idea of sample numbers. For example, if annual average rainfall for a region is 1000 mm and runoff ranges from 10% to 20% of annual rainfall, then discrete flow-interval sampling based on a 1-mm sampling interval would produce between 100 and 200 samples per year.

Selecting a Sampling Strategy

Based on the previous discussion of sampling strategy components and project considerations, we developed a flowchart to assist in selecting a general sampling strategy: flow-interval discrete, flow-interval composite, time-interval discrete, or time-interval composite (fig. 3). Following the flowchart and examining strategy advantages and disadvantages (table 3) along with project-specific considerations should result in the selection of an appropriate general sampling strategy based on project goals. Then based on that general strategy, the discussion below and data presented in tables 1, 2, and 4 will assist in selecting a specific strategy with an appropriate time or flow interval and discrete or composite sampling with a specific number of composite samples.

As previously stated, a compromise must be made between sample numbers (affected by sample timing, frequency, and type), equipment limitations, and measurement uncertainty. Table 4 is presented to illustrate this compromise and provide guidance for developing strategies for watersheds up to 1000 ha. The shaded cells in table 4 represent sampling strategies that fit both of the following criteria:

- Criterion 1 - The strategy will completely capture 90% of runoff events within a 24-bottle limitation - based on the number of discrete samples for the 90th percentile from table 1 and on the maximum runoff duration or runoff volume in table 2. This approximate criterion represents complete sampling for storm durations less than 2160 min (for time-interval sampling) and runoff depths less than 96 mm (for flow-interval sampling).

Table 3. Advantages and disadvantages of sampling interval types and collection options.

Time- or Flow-Interval Sampling	
**Time-Interval Sampling**	**Flow-Interval Sampling**
Advantages	Advantages
- Accurate at small time intervals	- More accurately measure storm loads
- Dependable (time measurement unlikely to fail)	- Relatively easy to choose proper flow interval
- Flow measurement not required to take samples	
Disadvantages	Disadvantages
- Difficult to choose proper time interval	- Flow measurement required to take samples
- Flow measurement needed to measure loads	- Flow control structure strongly recommended
	- Sampling will fail if flow measurement fails

Discrete and Composite Sampling	
**Discrete Sampling**	**Composite Sampling**
Advantages	Advantages
- Reduce sampling error	- Increase sampling duration/magnitude
- Capture within storm variability	- Decrease sample numbers
Disadvantages	Disadvantages
- Decrease sampling duration/magnitude	- Can increase sampling error
- Increase sample numbers	- Information on within storm variability is limited

- Criterion 2 - The strategy will on average measure loads within 20% of the true load – based on results of King and Harmel (2003) with similar errors presented by Miller et al. (2000).

The results represent strategy recommendations, not absolute answers, for sampling projects on watersheds less than 1000 ha. The results summarized in table 4 indicate that flow intervals less than 5 mm are preferred over larger flow intervals with the number of composite samples adjusted by project considerations. However, if average load errors less than 20% are desired, smaller flow intervals in the range of 1 to 3 mm are suggested. Results for flow-interval sampling do not change substantially as watershed size increases because volumetric depths normalize runoff volumes based on size. Whereas flow-interval strategies sample more frequently during higher flows, uniform time-interval strategies sample on equal time intervals making it difficult to achieve an acceptable uncertainty without excessive samples (tables 1 and 4). Therefore, variable time-intervals may be preferred over uniform time-intervals (especially if watershed hydrologic characteristics such as storm duration and watershed size are known on which to base the variable intervals).

Table 4. Suggested sampling strategies for watersheds less than 1000 ha (based on meeting criteria 1 and 2).

Time Interval		Composite Samples Per Bottle		
(min)	Discrete	2	4	6
5	2	2	2	2
10	2	2	2	2
15	2	2	2	**1 and 2**
30	2	2	**1 and 2**	1
60	2	**1 and 2**	1	1
120	**1 and 2**	1	1	1
180	1	1	1	1
300	1	1	1	1
360	1	1	1	1

Flow Interval		Composite Samples Per Bottle		
(mm)	Discrete	2	4	6
1.0	2	2	**1 and 2**	**1 and 2**
2.5	2	**1 and 2**	**1 and 2**	**1 and 2**
5.0	**1 and 2**	**1 and 2**	**1 and 2**	**1 and 2**
7.5	1	1	1	1
10.0	1	1	1	1
12.5	1	1	1	1
15.0	1	1	1	1

CONCLUSIONS

Storm water sampling projects with automated equipment require substantial investments of personnel time and funding to purchase and maintain sampling and flow monitoring equipment and to analyze collected samples. Because of this substantial investment, guidance on developing sound sampling strategies is needed. The objectives of this article are to present information on sampling strategy components and important considerations and to make general recommendations on developing appropriate project-specific, automated sampling strategies. The major sampling strategy components (minimum flow threshold, sampling interval, and sample type) and project-specific considerations (project goal, project resources, and watershed data) affect sample timing, frequency, costs, and errors and ultimately determine the success or failure of automated sampling strategies. Although, it is important to adequately consider all of these components and their interactions when designing a sampling strategy, several recommendations and considerations warrant special emphasis.

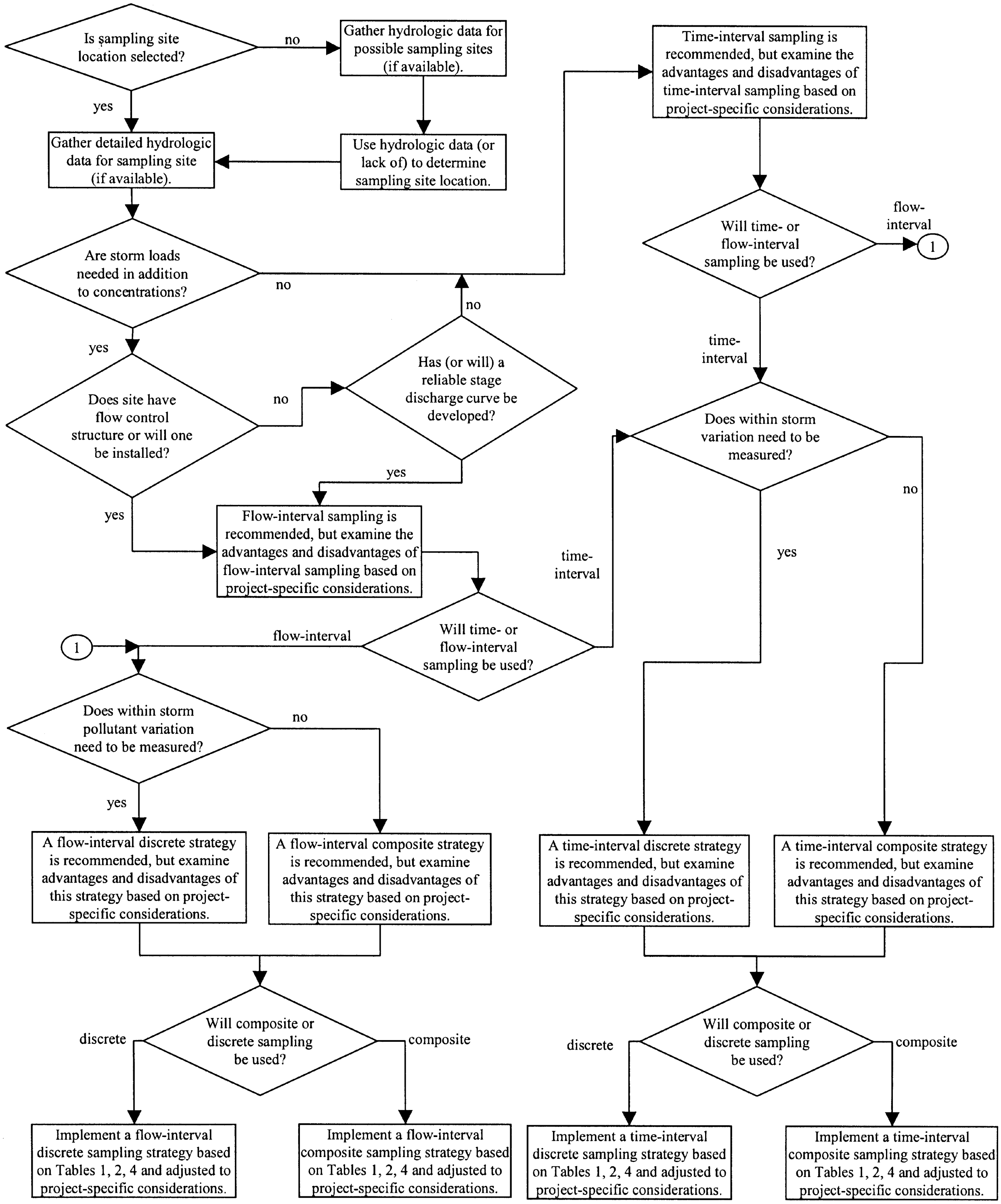

Figure 3. Flowchart for determining an appropriate general sampling strategy for automated storm water sampling of small watersheds.

Sampling Strategy Recommendations

- Minimum flow thresholds should be set such that even small increases in flow depth are sampled. On small watersheds ranging from 6 to 67 ha, minimum flow thresholds of 0.001 to 0.04 m^3/s are recommended (Harmel et al., 2002).
- Flow-interval sampling, especially at low intervals of 1 to 3 mm, generally produces less error than time-interval sampling because of more frequent sampling during higher flow.
- Time-interval sampling is simpler and less expensive when concentrations and not loads are needed. Variable time-intervals are recommended over uniform time-intervals if watershed hydrologic characteristics are known on which to base the variable intervals.
- If it is necessary to reduce the number of samples, composite sampling is recommended because it tends to introduce

less error than raising minimum flow thresholds or increasing sampling intervals, especially for flow-interval sampling (Miller et al., 2000; King and Harmel, 2003).
- If measurement of within-storm concentration change is unnecessary, 75 to 150 flow-interval samples of 100 to 200 mL can be composited into a single sample (16-L bottle capacity) with the resulting sample representing the EMC. This sampling strategy option reduces analysis costs and allows complete sampling of large runoff events.

Project Considerations

- As project goals are developed, it should be decided whether pollutant concentration data are adequate or if pollutant load data are also needed. Also, the necessity of measuring within-storm concentration changes must be addressed.
- If flow measurement is necessary and if the project budget allows, a hydraulic control structure should be installed to eliminate the need to develop a stage-discharge relationship. If this is not feasible, sampling sites should be located near established gaging stations or at culverts or concrete channels to take advantage of available data and established relationships.
- Frequent maintenance of automated samplers must be performed to limit malfunctions during storm events. Project resources must be adequate to cover this expensive and time-consuming task.
- Watershed hydrologic characteristics such as runoff volume, rainfall depth, and storm duration and intensity provide valuable information to guide development of sampling strategies.

Based on the sampling strategy components and project-specific considerations discussed, a process is presented to assist in designing automated storm water sampling strategies for small watersheds. The flowchart provides assistance in selecting a time- or flow-interval strategy with discrete or composite sampling (fig. 3). With the flowchart, advantages and disadvantages of each strategy (table 3), and project-specific considerations, selection of the appropriate sampling strategy can be confirmed. Then from that general strategy, data presented in tables 1, 2, and 4 can guide the selection of a specific strategy with an appropriate time or flow interval and discrete or composite sampling.

Acknowledgements

We would like to recognize the sampling work conducted by the Texas Institute of Applied Environmental Research in Stephenville, Texas, and led by Larry Hauck (Assistant Director of Environmental Science) and Anne McFarland (Research Scientist). Their work has resulted in the collection of an extensive data set and thoughtful insight on small watershed sampling issues. The field personnel of the USGS have been collecting a wide range of data on storm flows for many years and also deserve recognition for their dedication and efforts that have resulted in a vast body of knowledge on issues such as flood warning and storm water quality.

References

Claridge, G. G. C. 1974. Automated system for collecting water samples in proportion to stream flow rate. *New Zealand Journal of Science* 18: 289-296.

Dissmeyer, G. E. 1994. Evaluating the effectiveness of forestry best management practices in meeting water quality goals or standards. USDA Forest Service, Southern Region, Atlanta, Ga.

Haan, C. T. 1977. *Statistical Methods in Hydrology.* Ames, Iowa: The Iowa State Press.

Harmel, R. D., K. W. King, J. Wolfe, and H. A. Torbert. 2002. Minimum flow considerations for automated storm sampling on small watersheds. *Texas Journal of Science* 54(2): 177-188.

Izuno, F. T., R. W. Rice, R. M. Garcia, L. T. Capone, and D. Downey. 1998. Time versus flow composite water sampling for regulatory purposes in the Everglades Agricultural Area. *Applied Engineering in Agriculture* 14(3): 257-266.

King, K. W., and R. D. Harmel. 2003. Considerations in selecting a water quality sampling strategy. *Transactions of the ASAE* 46(1): 63-73.

McFarland, A., and L. Hauck. 2001. Strategies for monitoring nonpoint source runoff. TIAER Report No. 0115. December.

Miller, P. S., B. A. Engel, and R. H. Mohtar. 2000. Sampling theory and mass load estimation from watershed water quality data. ASAE Paper No. 003050. St. Joseph, Mich.: ASAE.

Novotny, V., and H. Olem. 1994. *Water Quality: Prevention, Identification, and Management of Diffuse Pollution.* New York: Van Nostrand Reinhold.

Rekolainen, S., M. Posch, J. Kamari, and P. Ekholm. 1991. Evaluation of the accuracy and precision of annual phosphorus load estimates from two agricultural basins in Finland. *J. of Hydrology* 128: 237-255.

Richards, R. P., and J. Holloway. 1987. Monte Carlo studies of sampling strategies for estimating tributary loads. *Water Resources Research* 23(10): 1939-1948.

Robertson, D. M., and E. D. Roerish. 1999. Influence of various water quality sampling strategies on load estimates for small streams. *Water Resources Research* 35(12): 3747-3759.

Shih, G., W. Abtew, and J. Obeysekera. 1994. Accuracy of nutrient runoff load calculations using time-composite sampling. *Transactions of ASAE* 37(2): 419-429.

Stone, K. C., P. G. Hunt, J. M. Novak, M. H. Johnson, and D. W. Watts. 2000. Flow-proportional, time-composited, and grab sample estimation of nitrogen export from an Eastern Coastal Plain watershed. *Transactions of ASAE* 43(2): 281-290.

Tate, K. W., R. A. Dahlgren, M. J. Singer, B. Allen-Diaz, and E. R. Atwill. 1999. Timing, frequency of sampling affect accuracy of water-quality monitoring. *California Agriculture* 53(6): 44-49.

United States Environmental Protection Agency. 1995. National water quality inventory 1994 report to Congress. USEPA 841-R-95-005. USEPA, Office of Water, Washington, D.C.

United States Environmental Protection Agency. 2000. National water quality inventory 1998 report to Congress. USEPA 841-R-00-001. USEPA, Office of Water, Washington, D.C.

Wells, F. C., W. J. Gibbons, and M. E. Dorsey. 1990. Guidelines for collection and field analysis of water-quality samples from streams in Texas. United States Geological Survey Open-File Report 90-127.
