# Supplementary material for: Watershed‐scale effects of tallgrass prairie reconstruction: 30‐Year trends in streamflow, nitrate, and sediment in Walnut Creek, Iowa
Source: J Environ Qual. 2026 Apr 5;55(2):e70174. doi: 10.1002/jeq2.70174 (PMC13051032; doi:10.1002/jeq2.70174)
Supplement: Supplementary file 1 — The Supporting Information details the analytical and collection methods used to obtain the streamflow, nitrate, and SSC data utilized in this study. Each of these datasets has also been included. Additionally, the Supporting Information contains the error metrics and residual plots from the WRTDSK models used to estimate daily nitrate and SSC concentrations. All annual values (i.e., annual yields, flow‐weighted concentrations, and average concentrations) have also been included. [file JEQ2-55-0-s001.zip › supplemental/analytical methods/ars_methods_discharge.pdf]

USDA/ARS Methods Catalog

**MethodID**	NSTL FM2
**Method Name**	Open Channel Flow Measurement of Water with Stream Bed Control
**Media**	water
**Method Type**	Field
**Method Subcategory**	Physical
**Method Source**	USDA, USGS
**Source Citation**	Brakensiek, D. L. et al. 1979. Field Methods for Research in Agricultural Hydrology. U.S. Department of Agriculture, Agriculture Handbook 224. Rantz S. E. 1982. Measurement and Computation of Streamflow. Geological Survey Water-Supply Paper 2175.
**Method Summary**	A calculated flow rate from stage and rating curve. The rating curve was developed from a series of stream discharge measurements performed over a range of stream depths.
**Instrument**	Stream Bed Control Measuring System
**Detection Limit Type**	
**DLNote**	
**Scope - Application**	
**Concentration Range**	
**Concentration Units**	
**Intereferences**	
**Precision Notes**	
**QA Requirements**	Monthly discharge measurements to develop rating curve shifts to correct for stream channel changes.
**Sampling Handling**	
**Max Holding Time**	
**Sample Prep Methods**	
**Link To Full Method**	NSTL Stream Gauge SOP.doc
**Method Contact**	USDA/ARS Kevin Cole, kevin.j.cole@ars.usda.gov

Analytes using this Method:

Analyte: Stream discharge MethodID: NSTL_FM2

Detection level: 0.003 cms

	Instrument	Matrix
**Accuracy**	5	
**Precision**	0.03	

False Positive Value: False Positive Value:

Accuracy/Precision Concetration Used:

Analyte: Discharge MethodID: NSTL_FM2

Detection level: 0.003 cms

	Instrument	Matrix
**Accuracy**	2	
**Precision**	0.003	

False Positive Value: False Positive Value:

Accuracy/Precision Concetration Used:
