# Supplementary material for: Watershed‐scale effects of tallgrass prairie reconstruction: 30‐Year trends in streamflow, nitrate, and sediment in Walnut Creek, Iowa
Source: J Environ Qual. 2026 Apr 5;55(2):e70174. doi: 10.1002/jeq2.70174 (PMC13051032; doi:10.1002/jeq2.70174)
Supplement: Supplementary file 1 — The Supporting Information details the analytical and collection methods used to obtain the streamflow, nitrate, and SSC data utilized in this study. Each of these datasets has also been included. Additionally, the Supporting Information contains the error metrics and residual plots from the WRTDSK models used to estimate daily nitrate and SSC concentrations. All annual values (i.e., annual yields, flow‐weighted concentrations, and average concentrations) have also been included. [file JEQ2-55-0-s001.zip › supplemental/analytical methods/ars_methods_nitrate.pdf]

USDA/ARS Methods Catalog

Field	Value
MethodID	NSTL FM10.1
Method Name	Manual Surface Water Sampling Transect Method EWI
Media	water
Method Type	Field
Method Subcategory	Sampling/Prep
Method Source	USGS
Source Citation	Edwards, T.K.; Glysson, G.D. 1999. Field Methods for Measurement of Fluvial Sediment. U.S.G.S., Techniques of Water-Resources Investigations, Book 3, Chapter C2
Method Summary	Collection of isokinetic, depth-integrated samples with equal width increment (EWI) methods which produces a discharge weighted concentrations oft he stream cross section.
Instrument	
Detection Limit Type	
DLNote	
Scope - Application	
Concentration Range	
Concentration Units	
Intereferences	
Precision Notes	
QA Requirements	
Sampling Handling	
Max Holding Time	
Sample Prep Methods	
Link To Full Method	
Method Contact	USDA/ARS Kevin Cole kevin.j.cole@ars.usda.gov

Analytes using this Method:

USDA/ARS Methods Catalog

**MethodID**	NSTL FM8
**Method Name**	Automated Surface Water Sampling
**Media**	water
**Method Type**	Field
**Method Subcategory**	Sampling/Preparation
**Method Source**	
**Source Citation**	
**Method Summary**	Automated waste water samplers adjacent to the stream under the control of a sampling program collect samples. The sample intake is along the side of the channel directly in the flow path in the lower part of the flow.
**Instrument**	Automated Water Sampler (Peristaltic Pump)
**Detection Limit Type**	
**DLNote**	
**Scope - Application**	
**Concentration Range**	
**Concentration Units**	
**Intereferences**	
**Precision Notes**	
**QA Requirements**	
**Sampling Handling**	Samples are transported to the laboratory at ambient temperature conditions, then temporarily stored in a refrigerator prior to analysis.
**Max Holding Time**	
**Sample Prep Methods**	
**Link To Full Method**	WC isco SOP.doc
**Method Contact**	USDA/ARS Kevin Cole, kevin.j.cole@ars.usda.gov

Analytes using this Method:

Analyte: **Automated water sample** MethodID: NSTL_FM8

Detection level: 200 ml

	Instrument	Matrix
**Accuracy**	0	
**Precision**	0	

False Positive Value: False Positive Value:

Accuracy/Precision Concetration Used:

Analyte: **Water sample** MethodID: NSTL_FM8

Detection level: 200 ml

	Instrument	Matrix
**Accuracy**	0	
**Precision**	0	

False Positive Value: False Positive Value:

Accuracy/Precision Concetration Used:

USDA/ARS Methods Catalog

**MethodID**	NSTL WQ1.1
**Method Name**	Determination of Nitrate/Nitrite by Flow Injection Analysis
**Media**	water
**Method Type**	Laboratory
**Method Subcategory**	Inorganic
**Method Source**	Lachat Instruments
**Source Citation**	Lachat Instruments, QuikChem method 10-107-04-1-E (Revision 5 Nov. 1992)
**Method Summary**	Passing the sample through a cadmium column reduces nitrate to nitrite. Reduced nitrate and original nitrite is determined by diazotizing with sulfanilamide and coupling with N-(1-naphthyl)-ethylenediamine dihydrochloride to form an azo dye that is measured at 520 nm; as described in QuikChem method 10-107-04-1-E.
**Instrument**	Automated Spectrophotometer
**Detection Limit Type**	RL
**DLNote**	
**Scope - Application**	This method is applicable to nitrate/nitrite determination in drinking, ground and surface water.
**Concentration Range**	1.0-5.0
**Concentration Units**	mg/L
**Intereferences**	residual chlorine; high concentrations of iron, copper, and other metals; oil and grease; and turbidity
**Precision Notes**	Precision and accuracy values were calculated using a certified external standard, the data was collected from the same sample ran on 5 separate days in a 30 day time period.
**QA Requirements**	daily calibration; external certified reference standard (ERA); check standard every 15 samples; matrix spikes (90-110% recovery; duplicates within 10% RPD
**Sampling Handling**	Samples are preserved at pH 2 with sulfuric acid and stored at 4 degrees C
**Max Holding Time**	30 days
**Sample Prep Methods**	
**Link To Full Method**	This method is proprietary. See www.lachatinsruments.com
**Method Contact**	USDA/ARS A. Morrow, amy.morrow@ars.usda.gov

Analytes using this Method:

Analyte: **Nitrate-N** MethodID: NSTL_WQ1.1

Detection level: 0.3 mg/L

	Instrument	Matrix
**Accuracy**	102.7	100.7
**Precision**	2.45	4.2

False Positive Value: False Positive Value:

Accuracy/Precision Concetration Used: 10 mg N/L
