# Supplementary material for: Watershed‐scale effects of tallgrass prairie reconstruction: 30‐Year trends in streamflow, nitrate, and sediment in Walnut Creek, Iowa
Source: J Environ Qual. 2026 Apr 5;55(2):e70174. doi: 10.1002/jeq2.70174 (PMC13051032; doi:10.1002/jeq2.70174)
Supplement: Supplementary file 1 — The Supporting Information details the analytical and collection methods used to obtain the streamflow, nitrate, and SSC data utilized in this study. Each of these datasets has also been included. Additionally, the Supporting Information contains the error metrics and residual plots from the WRTDSK models used to estimate daily nitrate and SSC concentrations. All annual values (i.e., annual yields, flow‐weighted concentrations, and average concentrations) have also been included. [file JEQ2-55-0-s001.zip › supplemental/analytical methods/ars_methods_SSC.pdf]

# USDA/ARS Methods Catalog

| | |
|---|---|
| **MethodID** | NSTL FM10.1 |
| **Method Name** | Manual Surface Water Sampling Transect Method EWI |
| **Media** | water |
| **Method Type** | Field |
| **Method Subcategory** | Sampling/Prep |
| **Method Source** | USGS |
| **Source Citation** | Edwards, T.K.; Glysson, G.D. 1999. Field Methods for Measurement of Fluvial Sediment. U.S.G.S., Techniques of Water-Resources Investigations, Book 3, Chapter C2 |
| **Method Summary** | Collection of isokinetic, depth-integrated samples with equal width increment (EWI) methods which produces a discharge weighted concentrations oft he stream cross section. |
| **Instrument** | |
| **Detection Limit Type** | |
| **DLNote** | |
| **Scope - Application** | |
| **Concentration Range** | |
| **Concentration Units** | |
| **Intereferences** | |
| **Precision Notes** | |
| **QA Requirements** | |
| **Sampling Handling** | |
| **Max Holding Time** | |
| **Sample Prep Methods** | |
| **Link To Full Method** | |
| **Method Contact** | USDA/ARS Kevin Cole kevin.j.cole@ars.usda.gov |

**Analytes using this Method:**

# USDA/ARS Methods Catalog

| | |
|---|---|
| **MethodID** | NSTL FM8 |
| **Method Name** | Automated Surface Water Sampling |
| **Media** | water |
| **Method Type** | Field |
| **Method Subcategory** | Sampling/Preparation |
| **Method Source** | |
| **Source Citation** | |
| **Method Summary** | Automated waste water samplers adjacent to the stream under the control of a sampling program collect samples. The sample intake is along the side of the channel directly in the flow path in the lower part of the flow. |
| **Instrument** | Automated Water Sampler (Peristaltic Pump) |
| **Detection Limit Type** | |
| **DLNote** | |
| **Scope - Application** | |
| **Concentration Range** | |
| **Concentration Units** | |
| **Intereferences** | |
| **Precision Notes** | |
| **QA Requirements** | |
| **Sampling Handling** | Samples are transported to the laboratory at ambient temperature conditions, then temporarily stored in a refrigerator prior to analysis. |
| **Max Holding Time** | |
| **Sample Prep Methods** | |
| **Link To Full Method** | WC isco SOP.doc |
| **Method Contact** | USDA/ARS Kevin Cole, kevin.j.cole@ars.usda.gov |

**Analytes using this Method:**

**Analyte:** **Automated water sample** MethodID: NSTL_FM8

Detection level: 200 ml

| | Instrument | Matrix |
|---|---|---|
| **Accuracy** | 0 | |
| **Precision** | 0 | |

False Positive Value: False Positive Value:

Accuracy/Precision Concetration Used:

**Analyte:** **Water sample** MethodID: NSTL_FM8

Detection level: 200 ml

| | Instrument | Matrix |
|---|---|---|
| **Accuracy** | 0 | |
| **Precision** | 0 | |

False Positive Value: False Positive Value:

Accuracy/Precision Concetration Used:

# USDA/ARS Methods Catalog

| | |
|---|---|
| **MethodID** | NSTL WO6 |
| **Method Name** | Suspended sediment in water |
| **Media** | water |
| **Method Type** | Laboratory |
| **Method Subcategory** | Physical |
| **Method Source** | ASTM |
| **Source Citation** | ASTM Standard D3977, 1997, "Standard Test Methods for Determining Sediment Concentration in Water Samples," ASTM International, West Conshohocken, PA, 1997 |
| **Method Summary** | A whole sample gravimetric analysis using filtration (glass fiber) for lower concentrations or evaporation for higher concentrations (>200 mg/l). |
| **Instrument** | |
| **Detection Limit Type** | |
| **DLNote** | |
| **Scope - Application** | |
| **Concentration Range** | 2 to 550000 |
| **Concentration Units** | mg/L |
| **Intereferences** | |
| **Precision Notes** | |
| **QA Requirements** | |
| **Sampling Handling** | |
| **Max Holding Time** | |
| **Sample Prep Methods** | |
| **Link To Full Method** | http://www.astm.org/cgi-bin/resolver.cgi?D3977 |
| **Method Contact** | USDA/ARS Kevin Cole, kevin.j.cole@ars.usda.gov |

**Analytes using this Method:**
