# Supplementary material for: Watershed‐scale effects of tallgrass prairie reconstruction: 30‐Year trends in streamflow, nitrate, and sediment in Walnut Creek, Iowa
Source: J Environ Qual. 2026 Apr 5;55(2):e70174. doi: 10.1002/jeq2.70174 (PMC13051032; doi:10.1002/jeq2.70174)
Supplement: Supplementary file 1 — The Supporting Information details the analytical and collection methods used to obtain the streamflow, nitrate, and SSC data utilized in this study. Each of these datasets has also been included. Additionally, the Supporting Information contains the error metrics and residual plots from the WRTDSK models used to estimate daily nitrate and SSC concentrations. All annual values (i.e., annual yields, flow‐weighted concentrations, and average concentrations) have also been included. [file JEQ2-55-0-s001.zip › supplemental/WRTDSK outputs/WNT1Nitrate_Plots.pdf]

Walnut Creek near Prairie City, IA
Nitrate

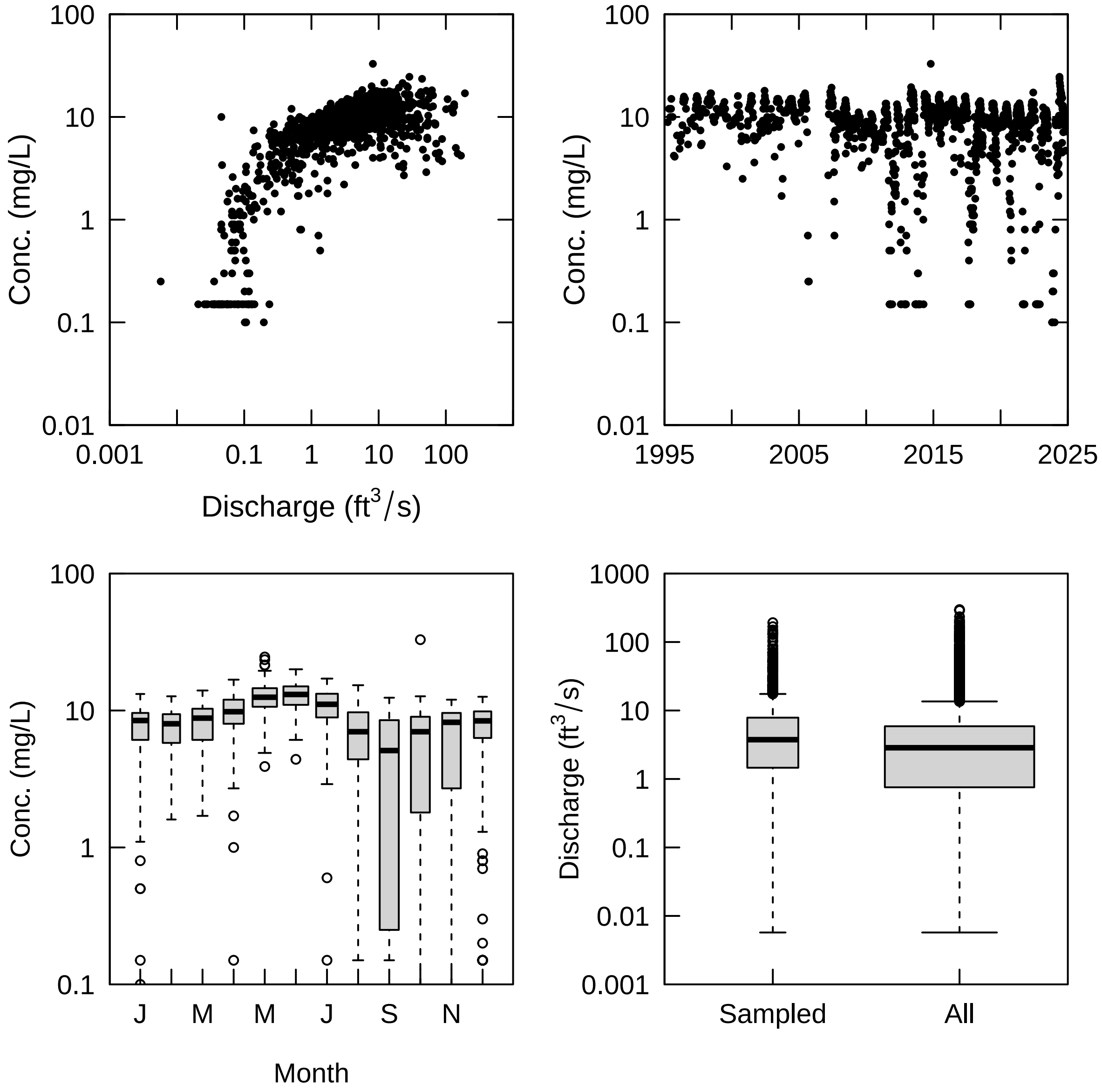

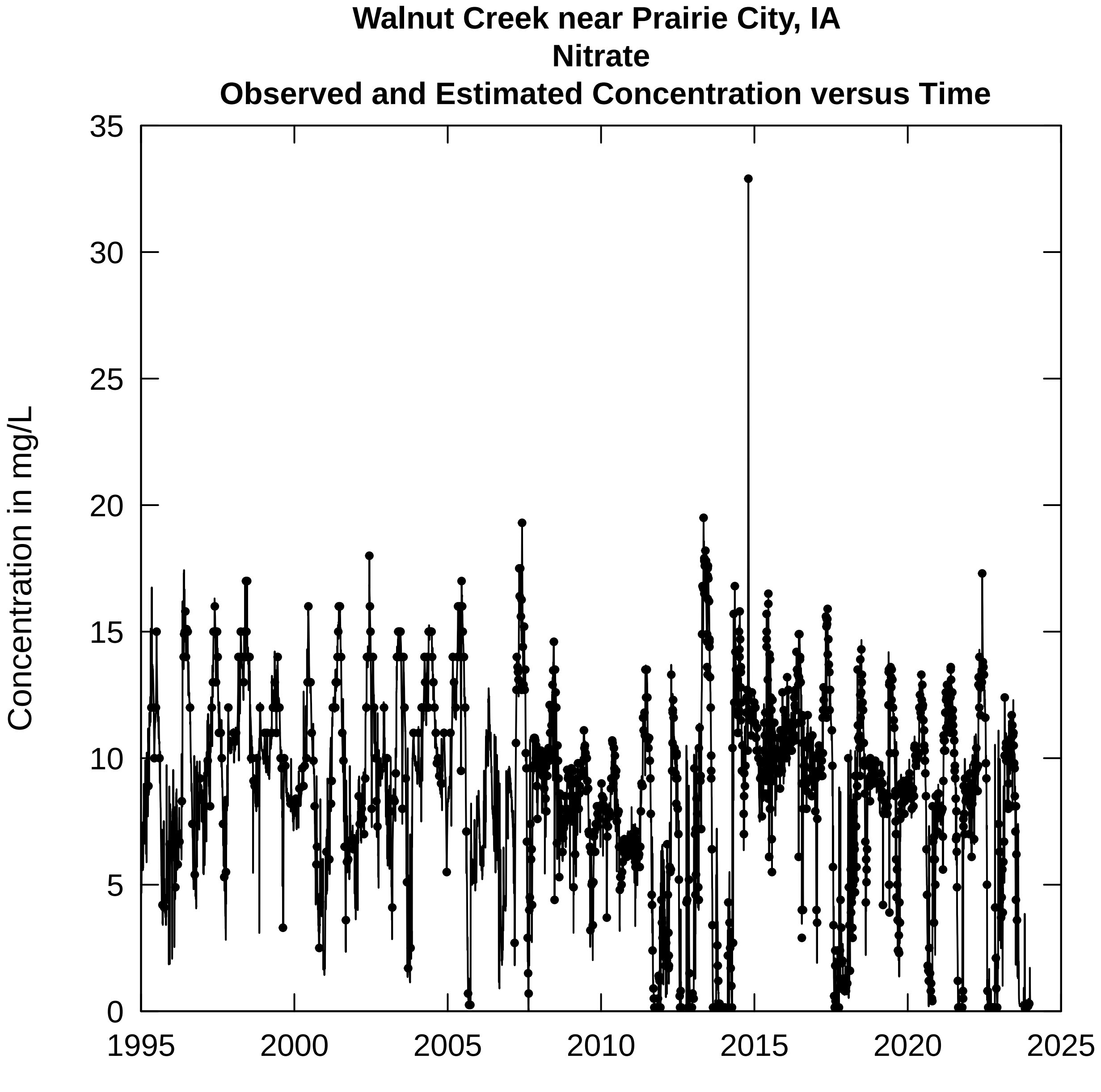
Walnut Creek near Prairie City, IA
Nitrate
Observed and Estimated Concentration versus Time
Concentration in mg/L
0
5
10
15
20
25
30
35
1995
2000
2005
2010
2015
2020
2025

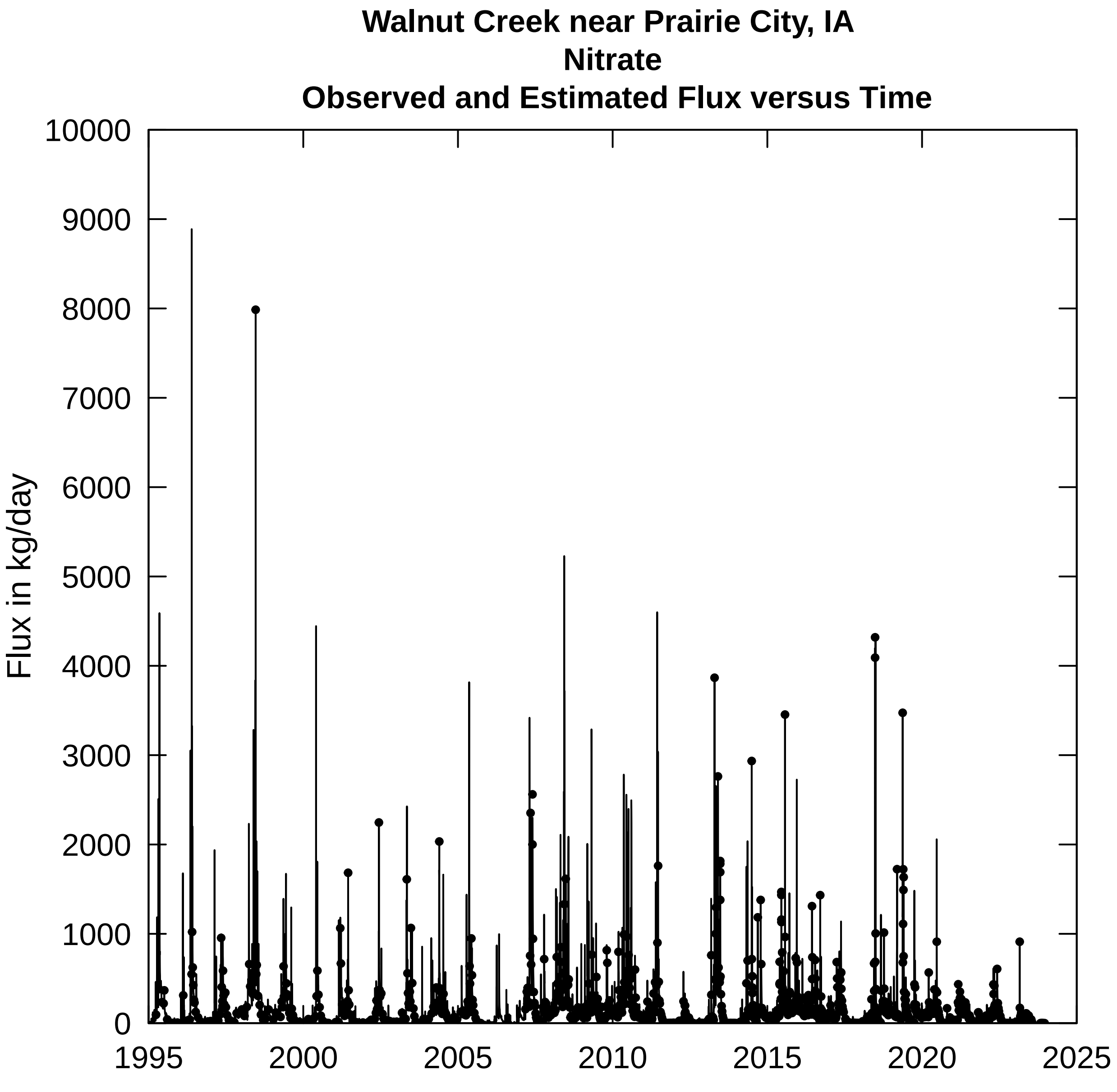

Walnut Creek near Prairie City, IA
Nitrate
Observed and Estimated Flux versus Time
Flux in kg/day
0
1000
2000
3000
4000
5000
6000
7000
8000
9000
10000
1995
2000
2005
2010
2015
2020
2025

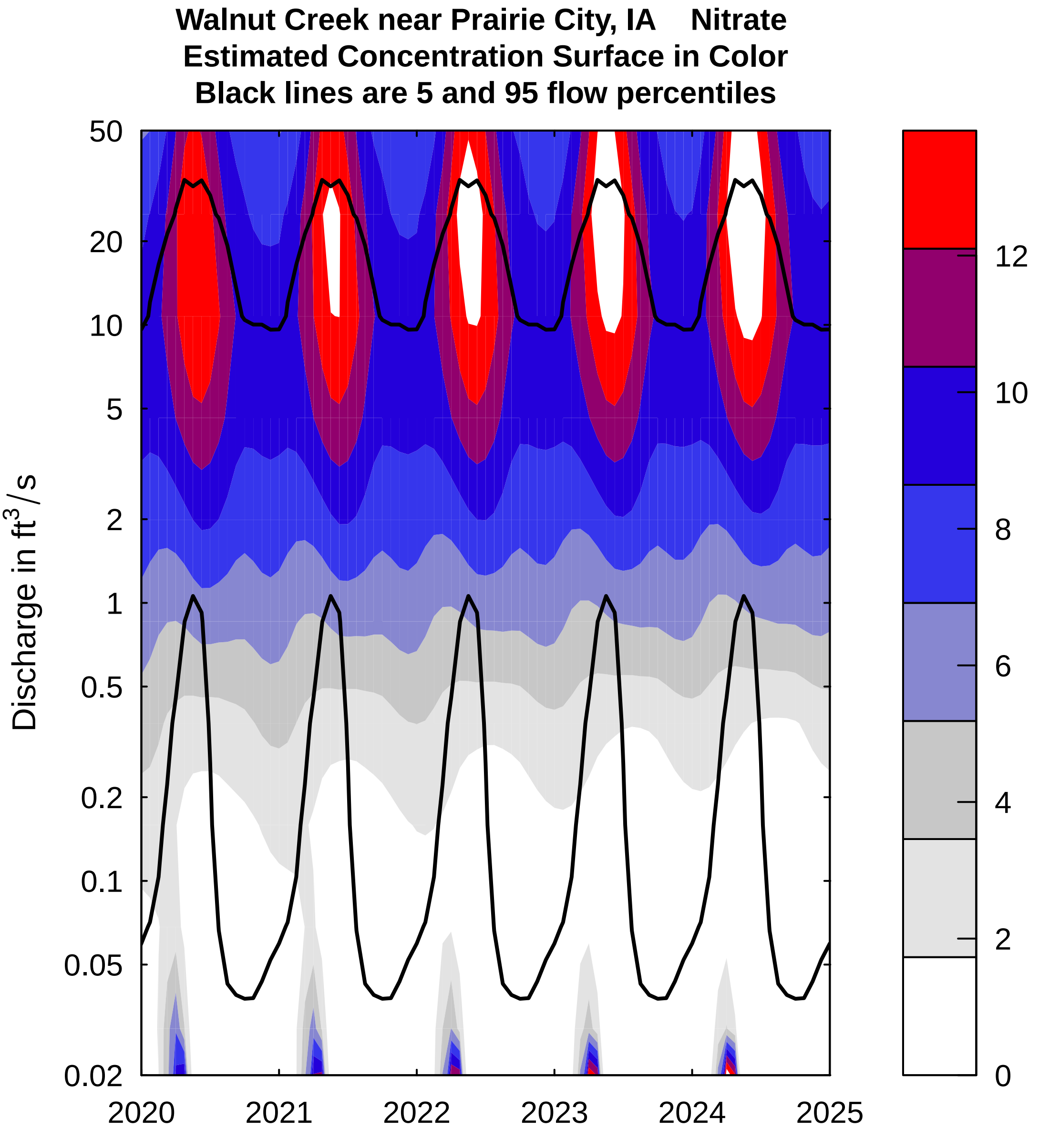

Walnut Creek near Prairie City, IA Nitrate
Estimated Concentration Surface in Color
Black lines are 5 and 95 flow percentiles
Discharge in ft^3/s
50
20
10
5
2
1
0.5
0.2
0.1
0.05
0.02
2020
2021
2022
2023
2024
2025
12
10
8
6
4
2
0

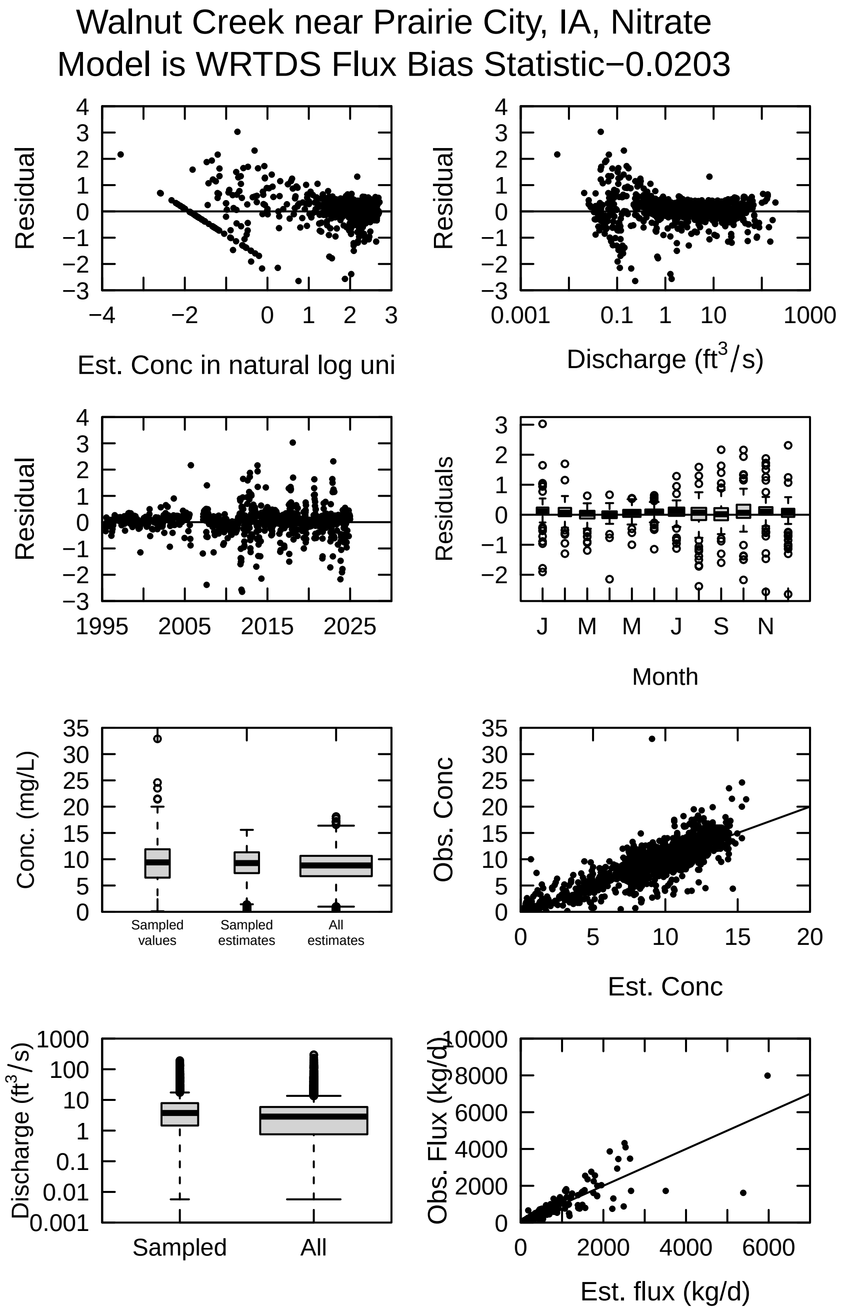

Walnut Creek near Prairie City, IA, Nitrate
Model is WRTDS Flux Bias Statistic−0.0203
Residual
Est. Conc in natural log uni
Residual
Discharge (ft3/s)
Residual
Residuals
J M M J S N
Month
Conc. (mg/L)
Sampled values
Sampled estimates
All estimates
Obs. Conc
Est. Conc
Discharge (ft3/s)
Sampled
All
Obs. Flux (kg/d)
Est. flux (kg/d)

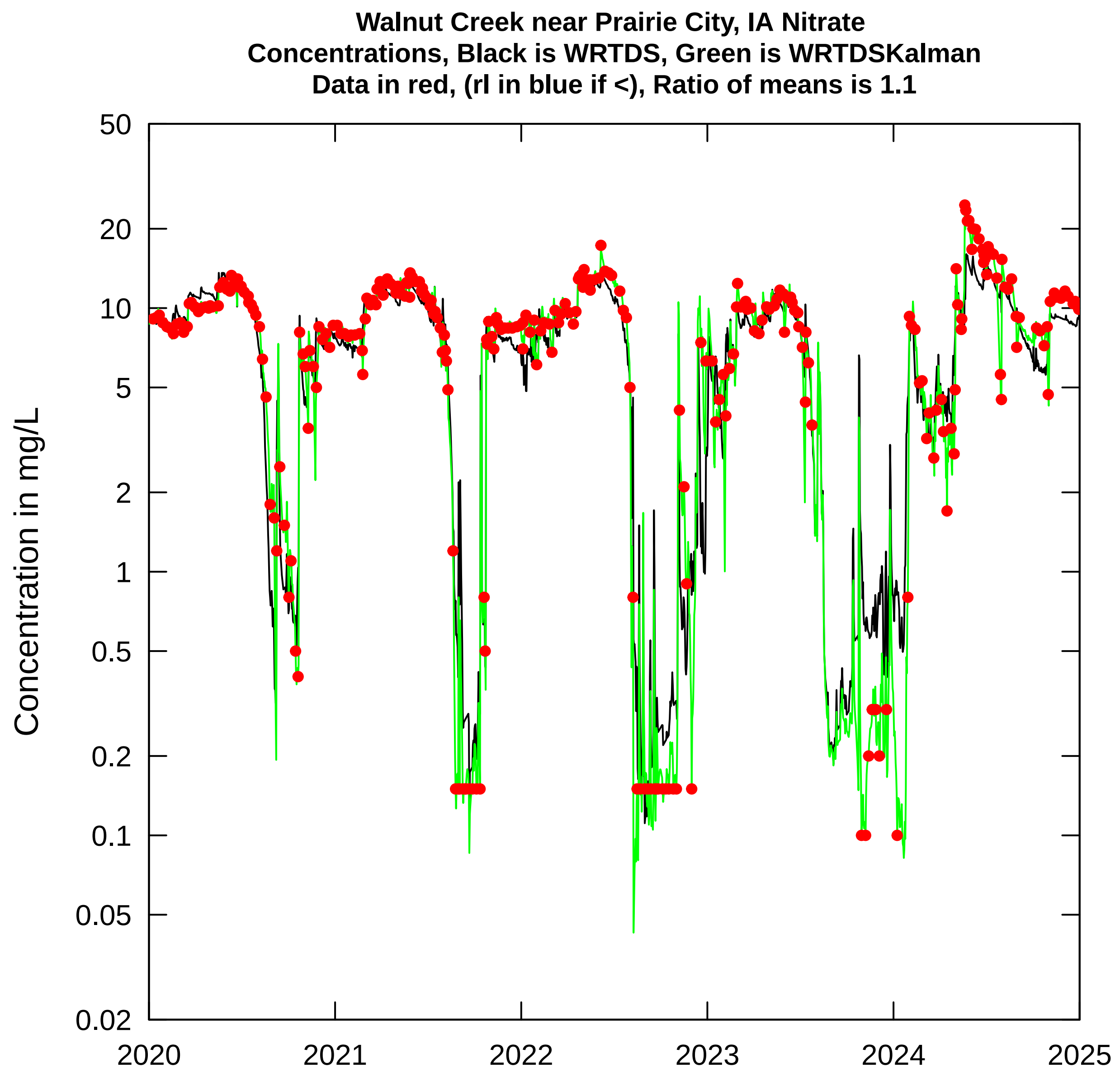
Walnut Creek near Prairie City, IA Nitrate
Concentrations, Black is WRTDS, Green is WRTDSKalman
Data in red, (rl in blue if <), Ratio of means is 1.1
Concentration in mg/L
50
20
10
5
2
1
0.5
0.2
0.1
0.05
0.02
2020
2021
2022
2023
2024
2025

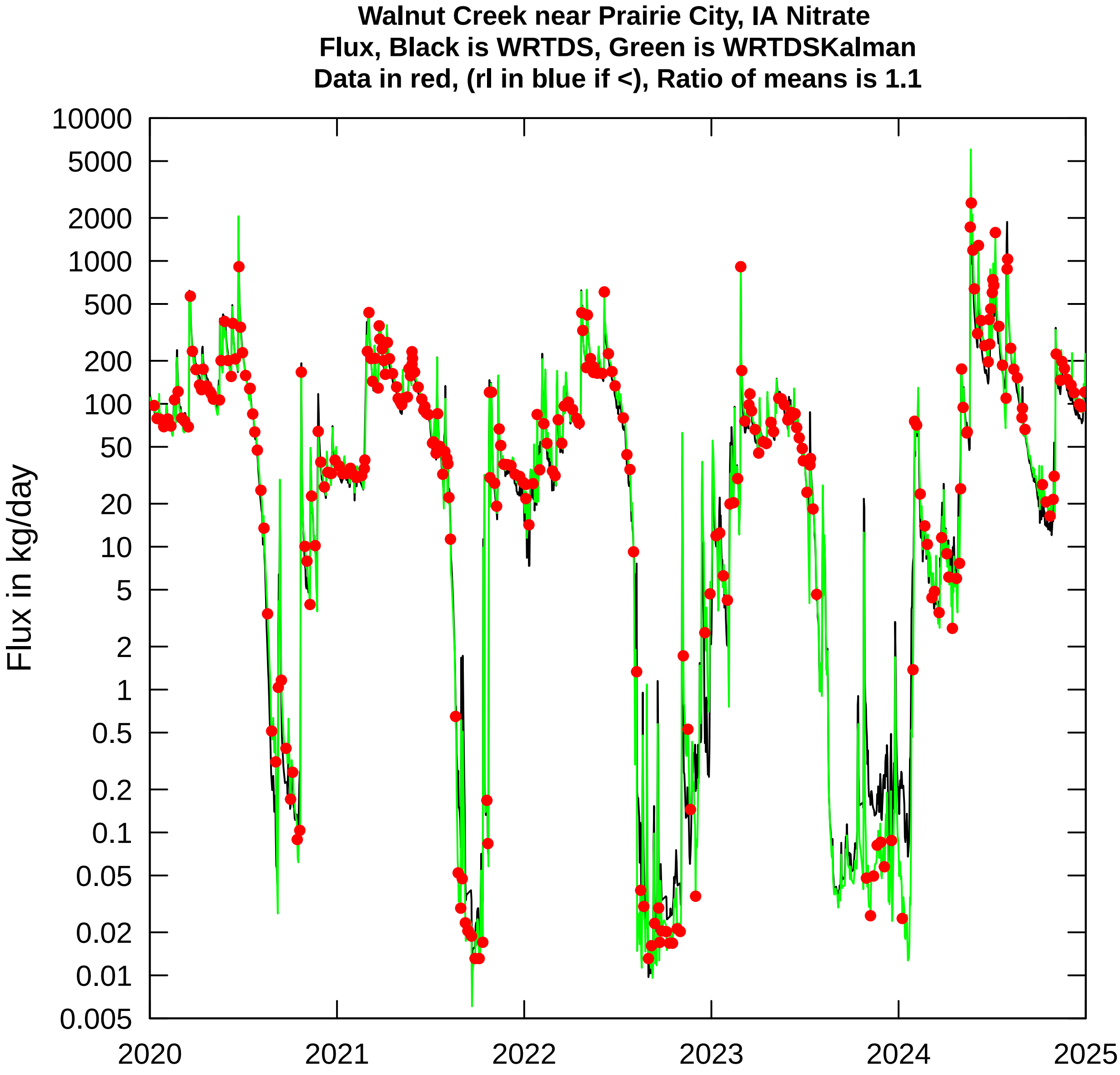
Walnut Creek near Prairie City, IA Nitrate
Flux, Black is WRTDS, Green is WRTDSKalman
Data in red, (rl in blue if <), Ratio of means is 1.1
Flux in kg/day
10000
5000
2000
1000
500
200
100
50
20
10
5
2
1
0.5
0.2
0.1
0.05
0.02
0.01
0.005
2020
2021
2022
2023
2024
2025
