# Supplementary material for: Watershed‐scale effects of tallgrass prairie reconstruction: 30‐Year trends in streamflow, nitrate, and sediment in Walnut Creek, Iowa
Source: J Environ Qual. 2026 Apr 5;55(2):e70174. doi: 10.1002/jeq2.70174 (PMC13051032; doi:10.1002/jeq2.70174)
Supplement: Supplementary file 1 — The Supporting Information details the analytical and collection methods used to obtain the streamflow, nitrate, and SSC data utilized in this study. Each of these datasets has also been included. Additionally, the Supporting Information contains the error metrics and residual plots from the WRTDSK models used to estimate daily nitrate and SSC concentrations. All annual values (i.e., annual yields, flow‐weighted concentrations, and average concentrations) have also been included. [file JEQ2-55-0-s001.zip › supplemental/WRTDSK outputs/WNT1SSC_Plots.pdf]

Walnut Creek near Prairie City, IA
Suspended Sediment Concentration

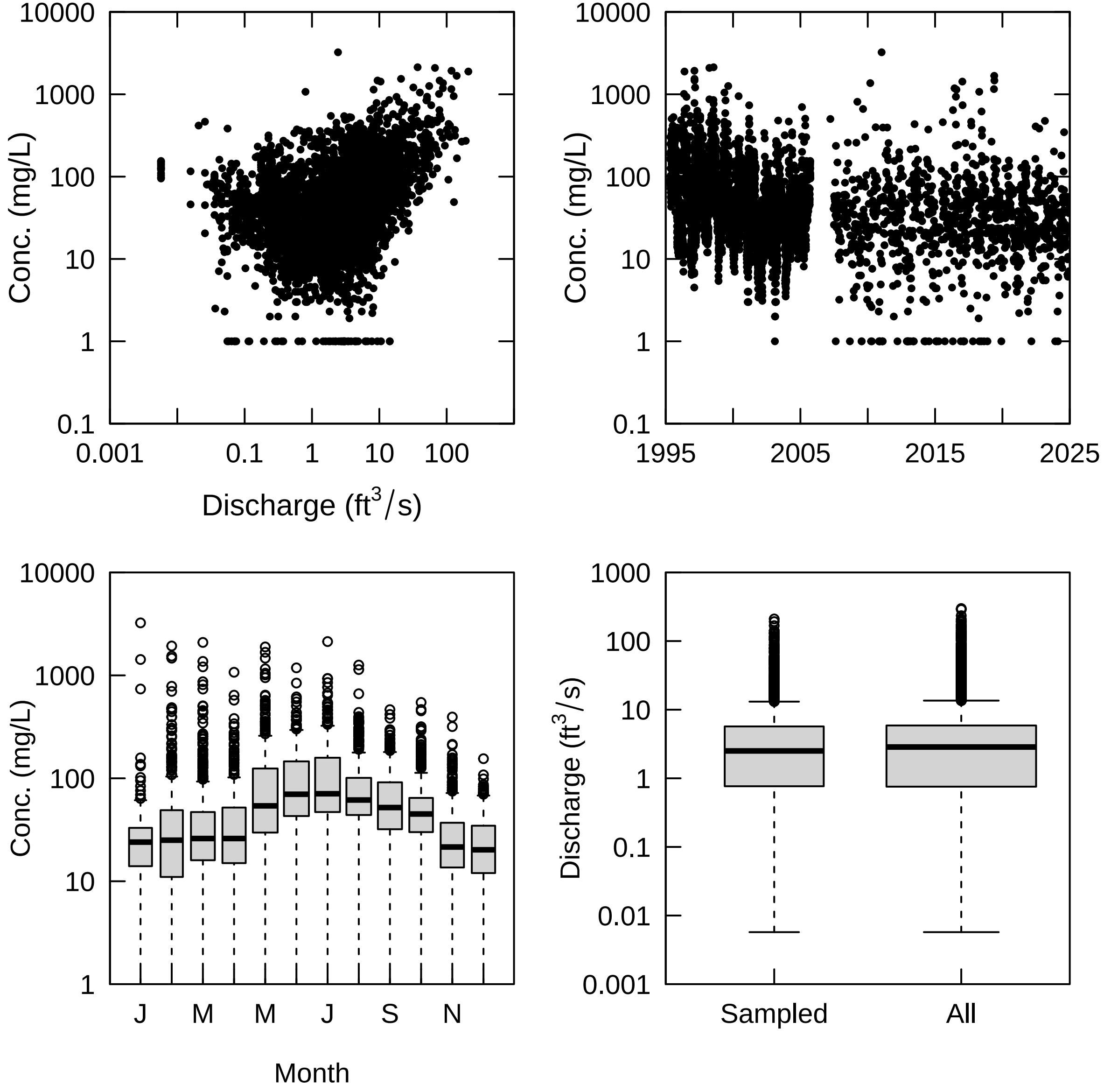

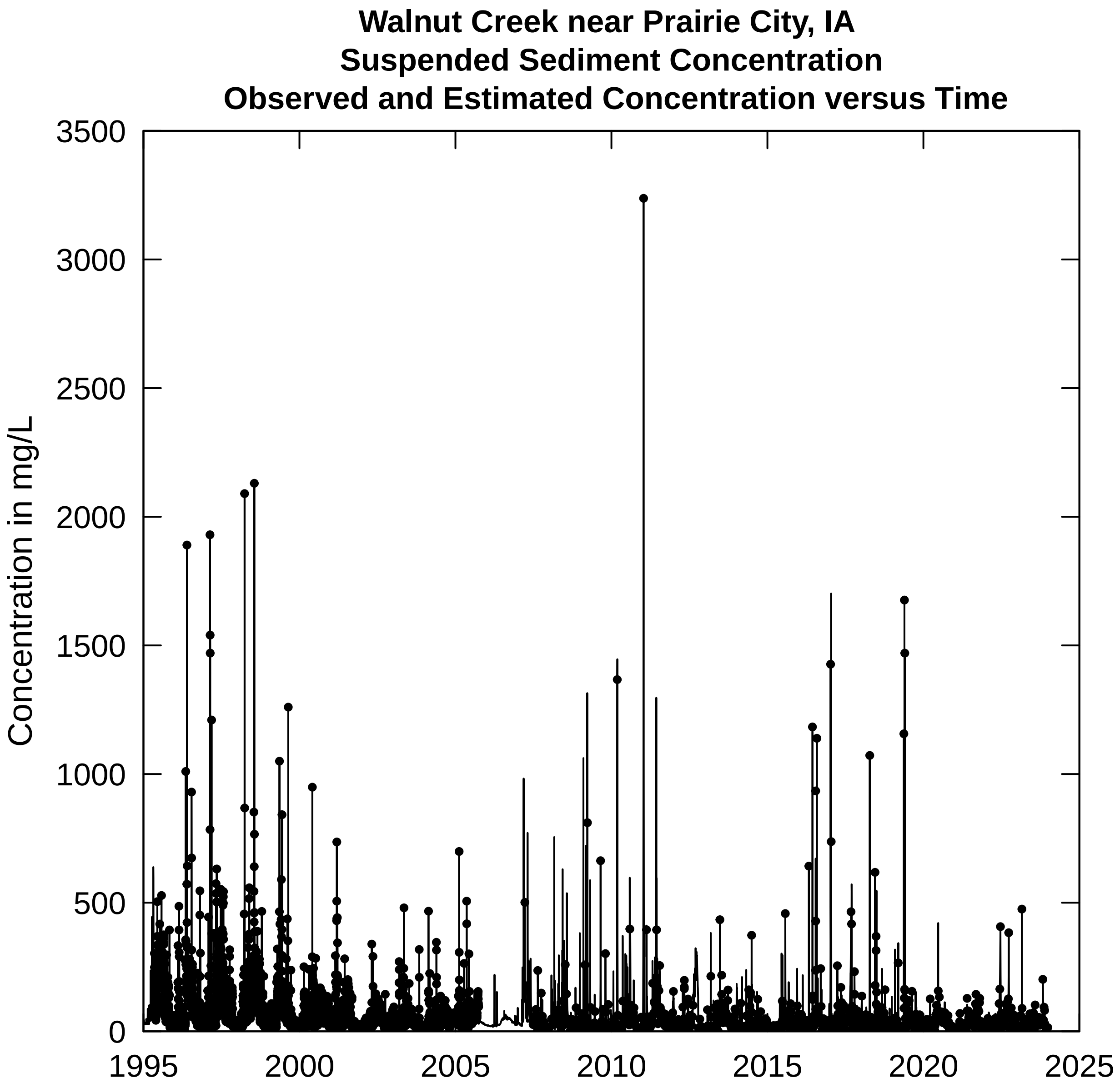
Walnut Creek near Prairie City, IA
Suspended Sediment Concentration
Observed and Estimated Concentration versus Time
Concentration in mg/L
3500
3000
2500
2000
1500
1000
500
0
1995
2000
2005
2010
2015
2020
2025

Walnut Creek near Prairie City, IA
Suspended Sediment Concentration
Observed and Estimated Flux versus Time

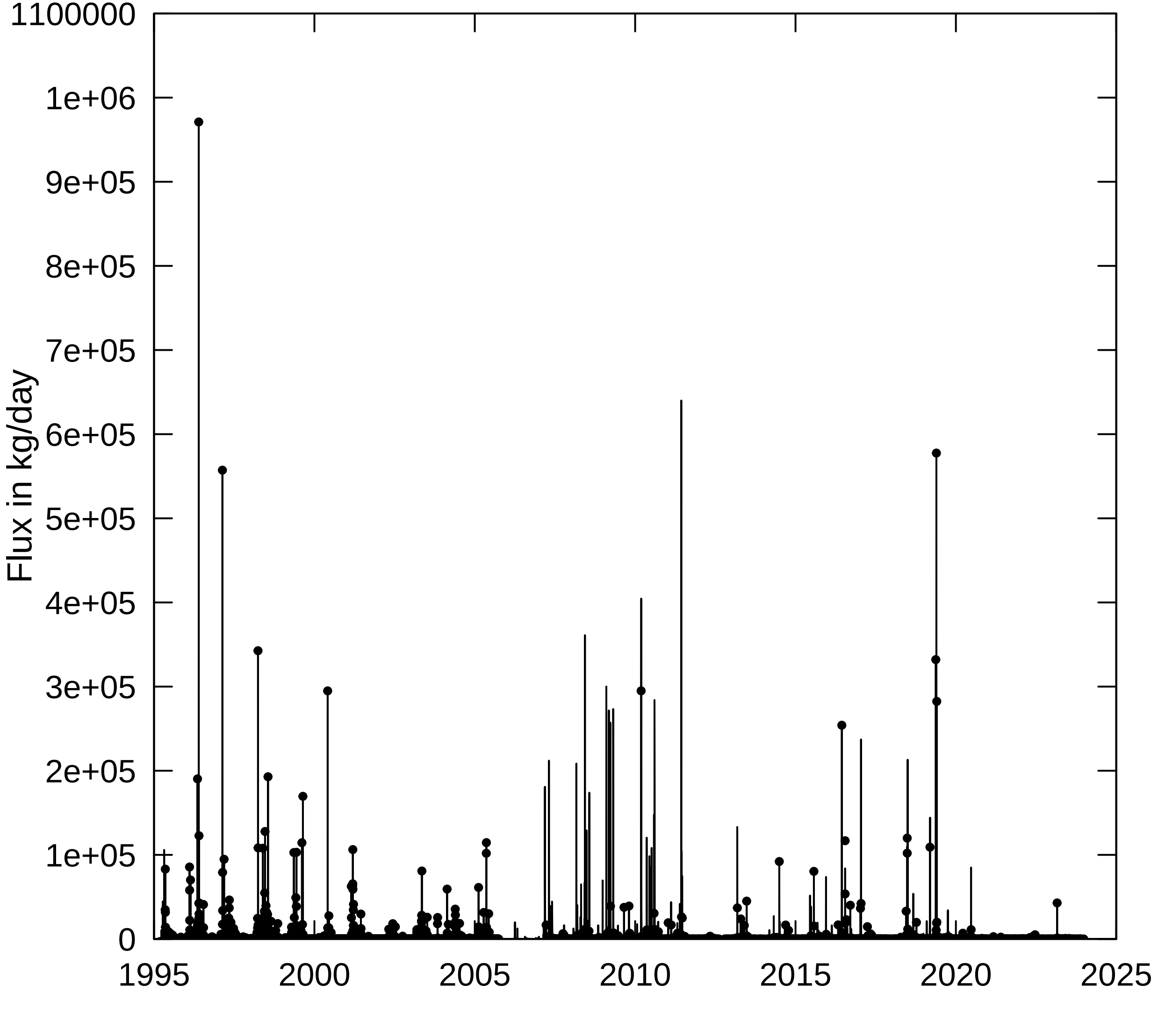

Walnut Creek near Prairie City, IA   Suspended Sediment Conc
Estimated Concentration Surface in Color
Black lines are 5 and 95 flow percentiles

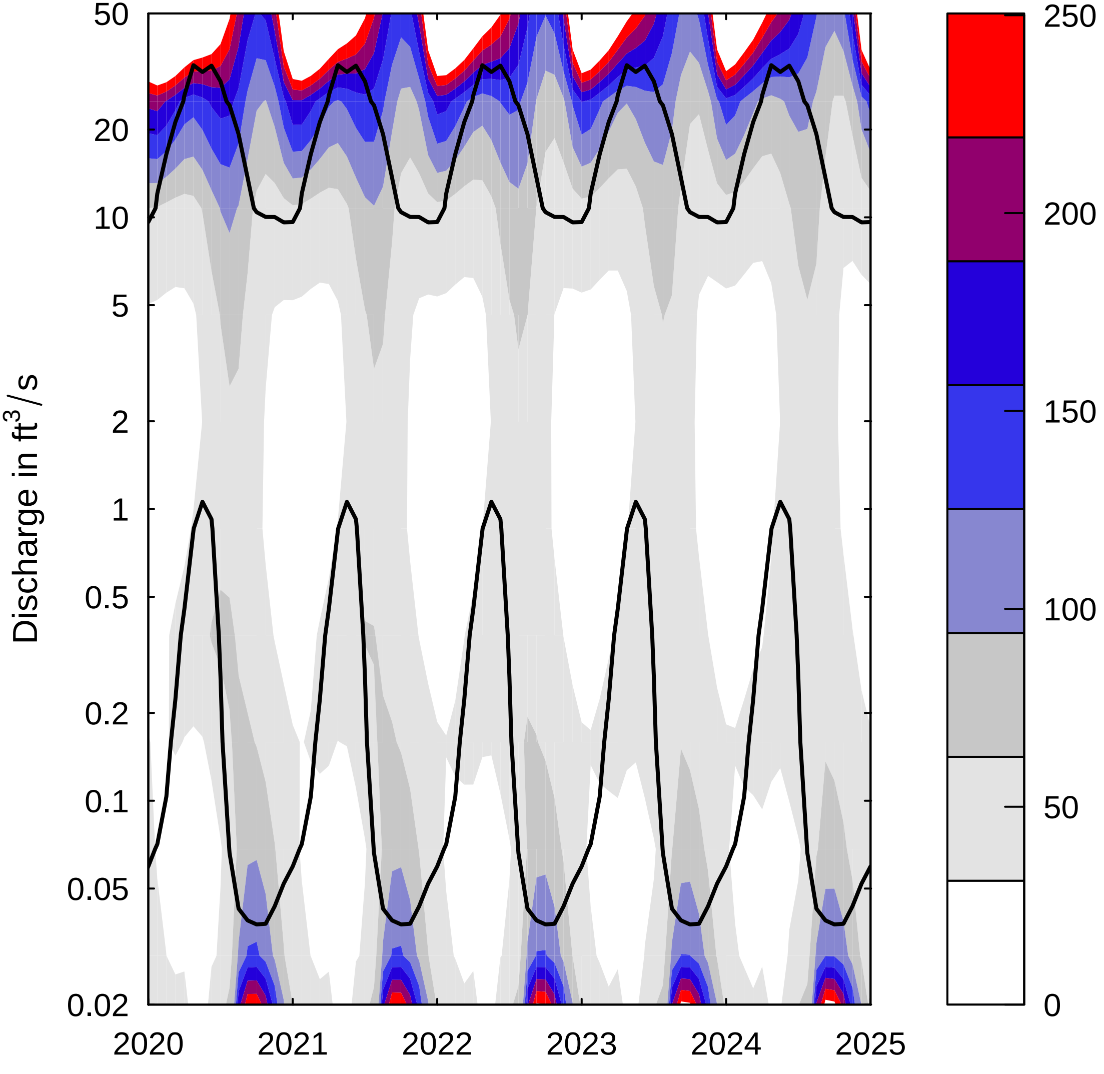

Walnut Creek near Prairie City, IA, Suspended Sediment Concentration
Model is WRTDS Flux Bias Statistic0.00939

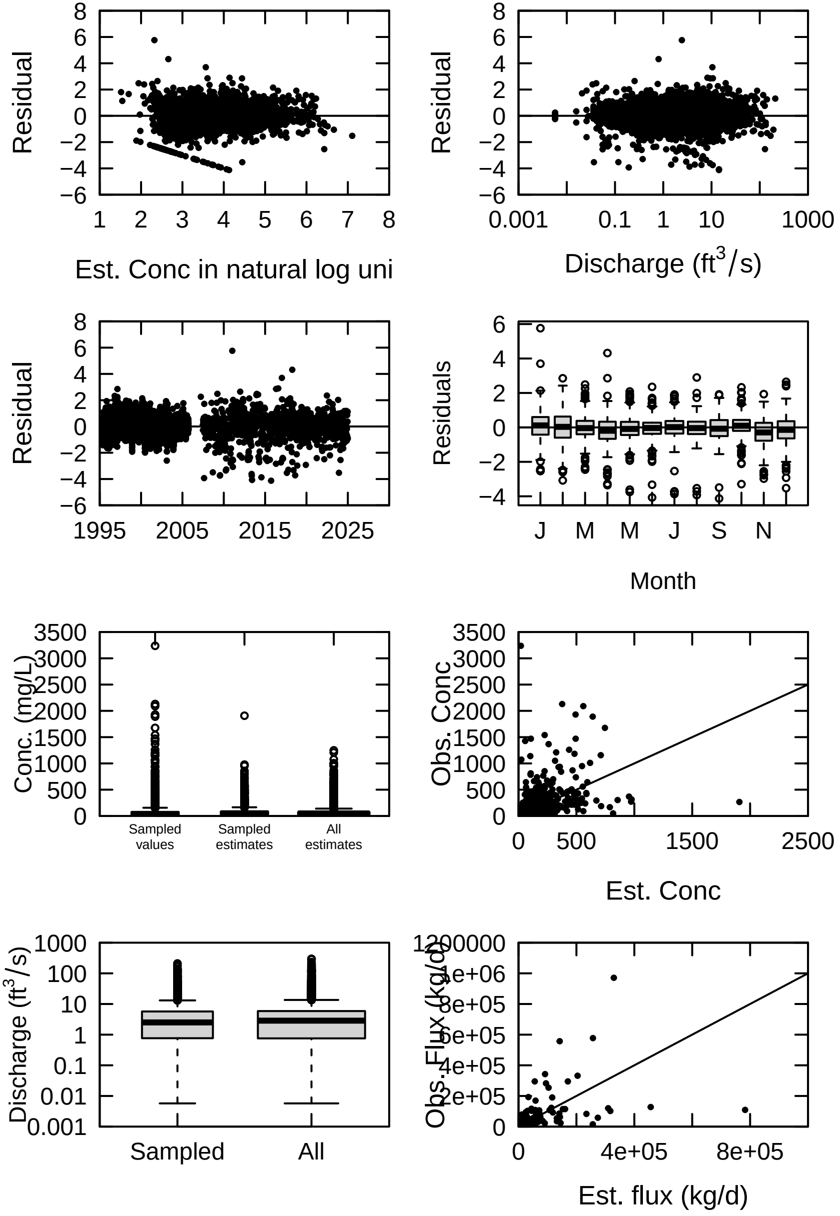

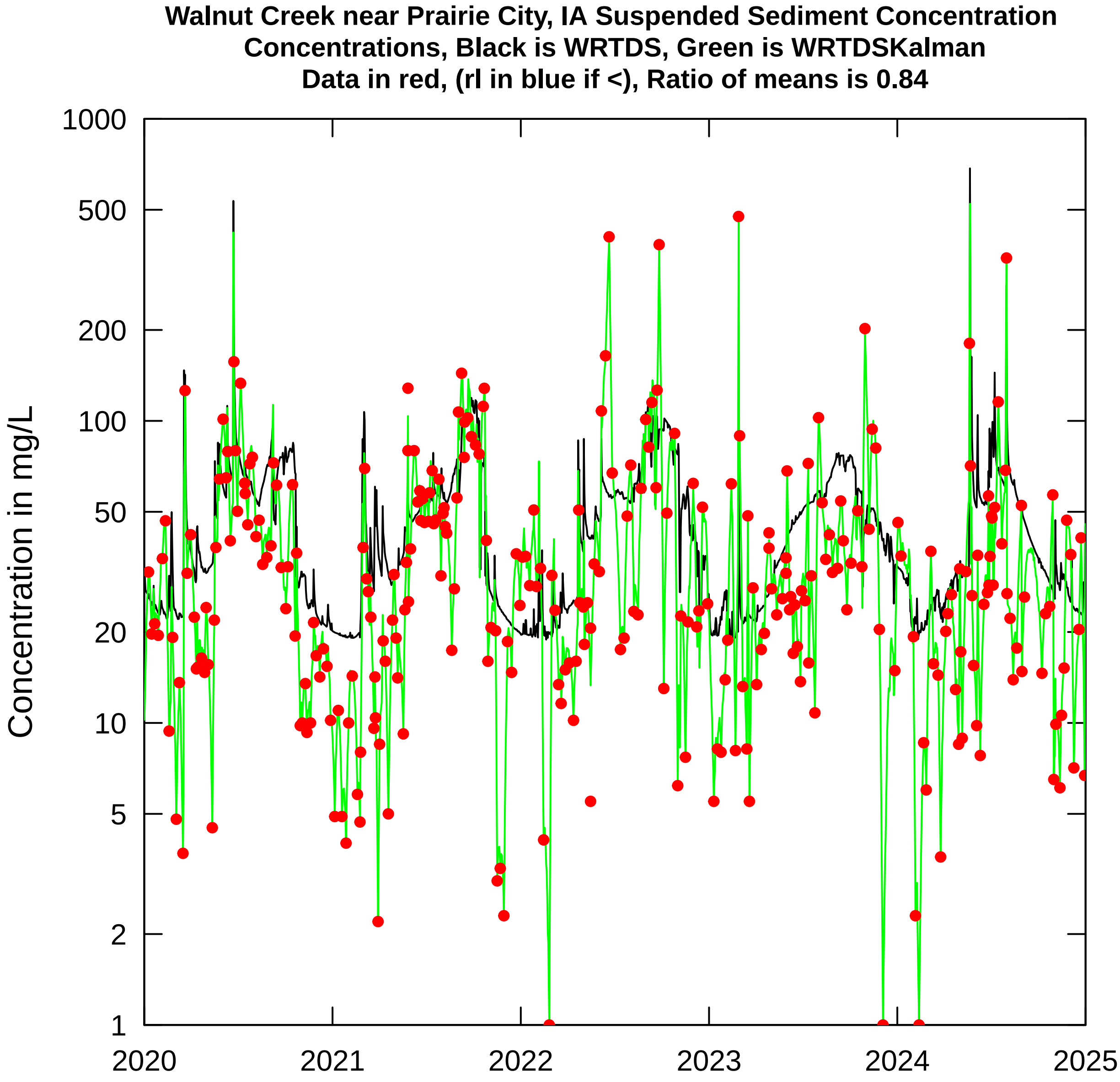
Walnut Creek near Prairie City, IA Suspended Sediment Concentration
Concentrations, Black is WRTDS, Green is WRTDSKalman
Data in red, (rl in blue if <), Ratio of means is 0.84
Concentration in mg/L
1000
500
200
100
50
20
10
5
2
1
2020
2021
2022
2023
2024
2025

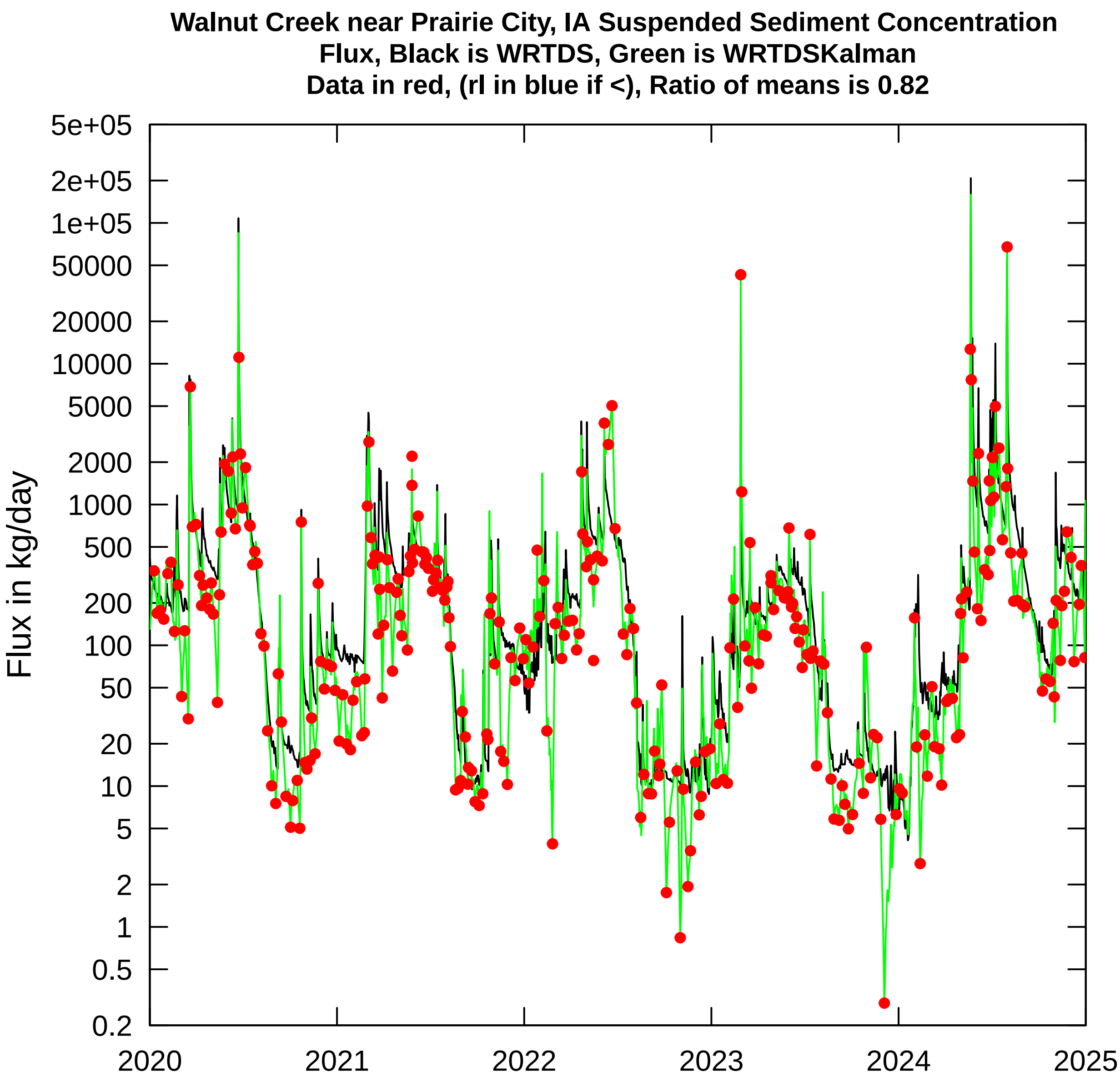
Walnut Creek near Prairie City, IA Suspended Sediment Concentration
Flux, Black is WRTDS, Green is WRTDSKalman
Data in red, (rl in blue if <), Ratio of means is 0.82
Flux in kg/day
5e+05
2e+05
1e+05
50000
20000
10000
5000
2000
1000
500
200
100
50
20
10
5
2
1
0.5
0.2
2020
2021
2022
2023
2024
2025
